# Brain Imaging Changes and Related Risk Factors of Cognitive Impairment in Patients With Heart Failure

**DOI:** 10.3389/fcvm.2021.838680

**Published:** 2022-01-26

**Authors:** Yangyang Jiang, Lei Wang, Ziwen Lu, Shiqi Chen, Yu Teng, Tong Li, Yang Li, Yingzhen Xie, Mingjing Zhao

**Affiliations:** ^1^Key Laboratory of Chinese Internal Medicine of Ministry of Education and Beijing, Dongzhimen Hospital Affiliated to Beijing University of Chinese Medicine, Beijing, China; ^2^Department of Encephalopathy, Dongzhimen Hospital Affiliated to Beijing University of Chinese Medicine, Beijing, China

**Keywords:** heart failure, cognitive impairment, brain imaging, risk factors, systematic review

## Abstract

**Background/Aims:**

To explore the imaging changes and related risk factors of heart failure (HF) patients with cognitive impairment (CI).

**Methods:**

A literature search was systematically carried out in PubMed, Web of Science, Embase, and Cochrane Library. In this systematic review, important relevant information was extracted according to the inclusion and exclusion criteria. The methodological quality was assessed by three scales according to the different study types.

**Results:**

Finally, 66 studies were included, involving 33,579 patients. In the imaging changes, the severity of medial temporal lobe atrophy (MTA) and the decrease of gray Matter (GM) volume were closely related to the cognitive decline. The reduction of cerebral blood flow (CBF) may be correlated with CI. However, the change of white matter (WM) volume was possibly independent of CI in HF patients. Specific risk factors were analyzed, and the data indicated that the increased levels of B-type natriuretic peptide (BNP)/N-terminal pro-B-type natriuretic peptide (NT-proBNP), and the comorbidities of HF, including atrial fibrillation (AF), diabetes mellitus (DM) and anemia were definitely correlated with CI in patients with HF, respectively. Certain studies had also obtained independent correlation results. Body mass index (BMI), depression and sleep disorder exhibited a tendency to be associated with CI. Low ejection fraction (EF) value (<30%) was inclined to be associated with the decline in cognitive function. However, no significant differences were noted between heart failure with preserved ejection fraction (HFpEF) and heart failure with reduced ejection fraction (HFrEF) in cognitive scores.

**Conclusion:**

BNP/NT-proBNP and the comorbidities of HF including AF, DM and anemia were inextricably correlated with CI in patients with HF, respectively. These parameters were independent factors. The severity of MTA, GM volume, BMI index, depression, sleep disorder, and low EF value (<30%) have a disposition to associated with CI. The reduction in the CBF volume may be related to CI, whereas the WM volume may not be associated with CI in HF patients. The present systematic review provides an important basis for the prevention and treatment of CI following HF.

## Introduction

Heart failure (HF), which is the end-stage manifestation of cardiovascular disease, is the leading cause of death and morbidity and its incidence increases rapidly along with age. HF not only damages the structure or function of the heart itself but also affects the physiological functions of multiple organs. Among them, cognitive impairment (CI) following HF is very common in clinical practice, and its prevalence rate is estimated to 31–85% according to previous investigations (1, 2). CI is associated with an array of poor outcomes in HF patients. Firstly, the presence of CI is strongly correlated with an increased risk of death and recurrent hospitalizations in the HF populations. Muradet al. (3) revealed in a longitudinal study that CI increased the risk of mortality in HF patients (HR 1.33, 95% CI 1.02–1.73). This was confirmed in subsequent studies (4, 5). Secondly, cognitive function is an important factor affecting the quality of life in HF patients. Elderly patients with HF may have worse self-care behaviors due to the development of CI, such as inability to receive their medications on time, dietary restrictions, and lifestyle changes, which will damage patient ability to self-care, and finally reduce the quality of life (6–8). Thirdly, the decline in cognitive function may also affect the clinical symptoms of HF. Memory loss, deterioration of quality of life, and poor executive function are more likely to increase the risk of adverse events (9). Therefore, the symptoms of HF and the deterioration of cognitive function promote and influence each other.

HF patients with CI demonstrated cognitive disorders in attention, orientation, episodic memory, visuospatial function, executive function, and other aspects. In the early stage of HF, patients may be more likely to show a decline in executive function, attention, and visuospatial ability, while the memory function may be slightly impaired (10). With the progression of the disease, the cognitive functions of patients with HF will decline to varying degrees and may develop into Alzheimer's disease (AD) or vascular dementia (VD) in the later stage (11).

Therefore, CI has negative effects on the prognosis of HF patients. Although its etiology and pathological mechanism are not completely clear, it is undeniable that CI following HF is caused due to the interaction of multiple factors. The decrease in the cerebral blood flow (CBF) or the changes in brain structure may be the pathological basis of CI in HF patients (12). In addition, decreased ejection fraction (EF), and comorbidities such as depression, atrial fibrillation (AF), and diabetes mellitus (DM) are also risk factors affecting cognitive function in HF patients (13). However, to the best of our knowledge, no systematic summary has been performed on brain imaging and on the effect of multiple related factors of CI following HF. The present article reviews the imaging changes and risk factors of CI in patients with HF so as to provide the basis for subsequent prevention and treatment.

## Methods

### Search Strategy

The search was applied to four databases including PubMed, Embase, Web of Science and Cochrane Library (the publishing time ranged from the inception through April 21, 2021). The search strategy used the following general terms as the mesh or the free terms: The search strategy used the search terms “heart failure,” “cognitive impairment,” “brain imaging,” “risk factors,” “cerebral blood flow,” “hippocampus,” “gray matters,” “temporal lobe,” “white matter,” “ejection fraction,” “body mass index,” “electrolyte,” “depressed,” “atrial fibrillation,” “diabetes,” “anemia,” “sleep disorders,” “BNP,” and their common synonyms. The specific retrieval strategy is described in the Supplementary Material 1.

### Inclusion and Exclusion Criteria

The inclusion criteria of the studies were as follows: (1) Participants: Patients with clinically diagnosed HF were included, regardless of their race or nationality, with or without the control group; (2) Method of research: Cohort studies, case-control studies, cross-sectional studies, and intervention studies were included; (3) Detection of indices: Imaging on CBF, hippocampus, temporal lobe, gray matter, and white matter; the risk factors included EF, BMI index, and electrolyte, BNP, and NT-proBNP levels; the comorbid diseases included depression, DM, AF, anemia, and sleep disorders; (4) Statistical methods: Correlation analyses were performed between detection indices and cognitive function in HF patients.

It should be noted that certain studies were included, although no correlation analysis was performed in the present systematic review. These studies researched the differences in cognitive function among different types of HF patients, and the types were distinguished by EF. Therefore, these studies can be regarded as the effects of EF on the cognitive function of HF patients.

The exclusion criteria were as follows: (1) non-clinical studies; (2) articles with incomplete information; (3) reviews, meta-analyses, and corresponding/conference abstracts.

### Data Extraction

The data were extracted independently by two evaluators. In case of a dispute, this was resolved following discussion by both parties or decided by the third evaluator. The data extracted included the first author's name, publication time, number of cases, patient characteristics (age, gender, type of HF, and cardiac function grade), imaging detection methods, cognitive function detect methods, and main results. The lack of information was supplemented by contacting the author by telephone or email.

### Quality Assessment

The agency for Healthcare Research and Quality (AHRQ) was used to assess the quality of cross-sectional studies (14). It included 11 items, such as defining the source of information, listing inclusion and exclusion criteria, and indicating the period used for identifying patients, which answered with “yes,” “no,” and “not clear.” The answer to “yes” scored 1 point, whereas the answer to “no or not clear” 0 points. A score of 0–3 points was indicative of low quality, of 4–7 points of medium quality, and of 8–11 points of high quality (15). The Newcastle–Ottawa scale (NOS) was used to assess the quality of cohort and case-control studies (16). The scale compared eight items from three aspects of study population selection, comparability between groups, and measurement of exposure factors. Each study scored 0–9 points. A score of 0–4 points was indicative of low quality, of 5–6 points of medium quality, and of 7–9 points of high quality. The Methodological Index for Non-randomized Studies (MINORS) was used to conduct the quality evaluation for the self-control studies (17). It includes 8 items, such as clear goals, continuous patient inclusion, prospective data collection, and unbiased assessment of the study endpoint. Each item received a score from 0–2 points, and the total score was 24 points. 0–8 points were indicative of low quality, 9–16 points of medium quality, and 17–24 points of high quality.

### Summary Analysis

A qualitative synthesis was adopted for this systematic review.

## Results

### Literature Search Results

**Figure 1:**
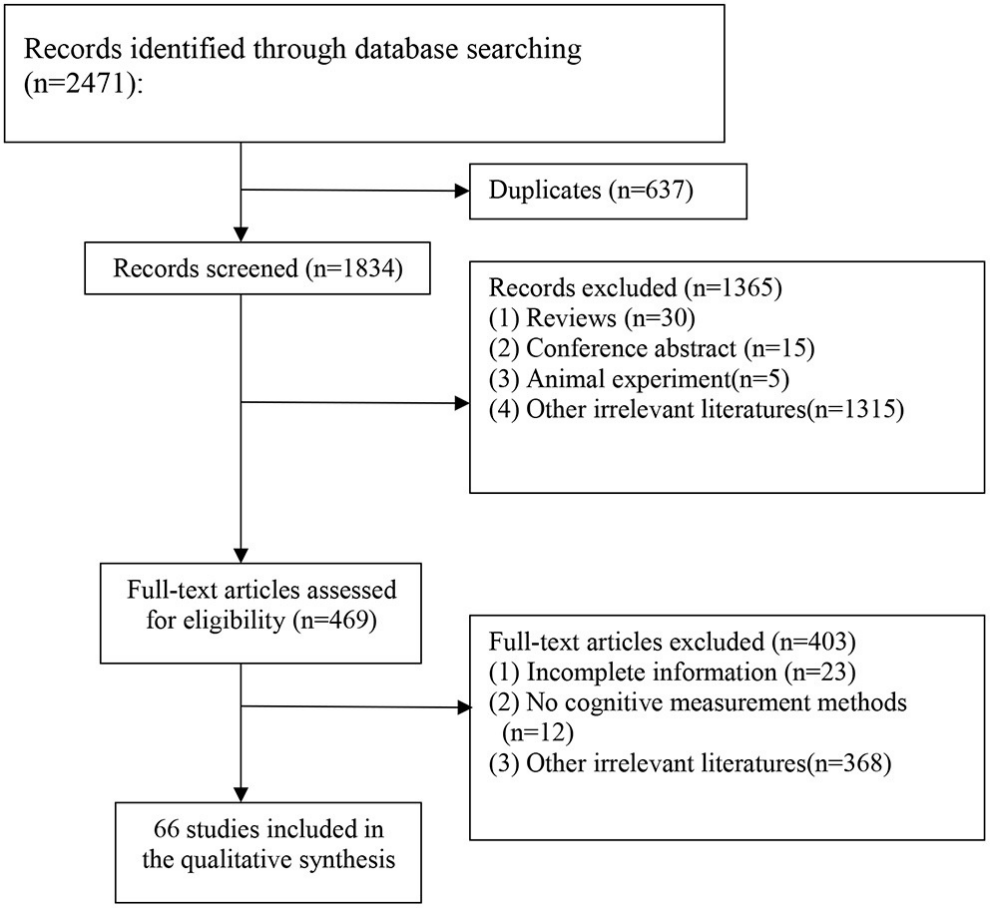
Flow chart of searching and screening studies.

A total of 2,471 records were identified from four electronic databases, and 637 duplicate articles were removed. Following evaluation of titles and abstracts, 1,365 articles were excluded. A total of 403 articles were excluded of the 469 remaining articles, following investigation of the full text. Finally, 66 studies (18–83) were included in the analysis, involving a total of 33,579 patients. The flow chart (Figure 1) indicates the search process and study selection.

### Study Characteristics

The study and patient characteristics of the included studies are shown in Tables 1–**12**. A total of eight studies (18–25) described the relationship between CBF and cognitive function in HF patients, whereas three studies (26–28) described the relationship between MTA and cognitive function in HF patients, and two studies (29, 30) described the relationship between GM changes and cognitive function in HF patients. Moreover, four studies (26–28, 31) described the relationship between WM changes and cognitive function in HF patients, whereas 16 studies (32–47) described the relationship between EF and cognitive function in HF patients, and seven studies (24, 37, 40, 48–51) described the relationship between BMI and cognitive function in HF patients. A total of five studies (39, 52–55) described the relationship between electrolyte levels and cognitive function in HF patients, whereas six studies (41, 46, 57, 81–83) described the relationship between BNP/NT-proBNP levels and cognitive function in HF patients, and 15 studies (23, 42, 44, 48, 56–66) described the relationship between depression and cognitive function in HF patients. In addition, five studies (21, 67–70) described the relationship between AF and cognitive function in HF patients, six studies (49, 55, 61, 69, 71, 72) assessed the relationship between DM and cognitive function in HF patients, six studies (40, 55, 59, 63, 71, 73) evaluated the relationship between anemia and cognitive function in HF patients, and eight studies (32, 74–80) described the relationship between sleep disorder and cognitive function in HF patients.

**Table 1:** The association between the changes noted in the CBF volume and the cognitive function in patients with HF.

**References**	**Study design**	**Participants**	**Mean age**	**Male %**	**Type of HF**	**Methods of CBF**	**Evaluation of cognitive function**	**Study results**
Leeuwis et al. (18)	Cross-sectional study	124 HF 75 COD 127 Possible VCI 113 Controls	HF 68.7 ± 9.9 COD 65.1 ± 7.5 VCI 68.3 ± 8.7 Controls 65.6 ± 7.1	62.4%	Not specified	3.0-Tesla-MRI pCASL	MMSE, VAT, RAVLT, TMTa, DLST, SCWT, TMTb	The study did not identify an association between CBF and CI.
Suzuki et al. (19)	Cross-sectional study	40 stage B HF 40 stage C HF	stage B 65.0 ± 10.9 stage C 66.8 ± 8.9	72.5%	Not specified	1.5-Tesla-MRI	MMSE, WMS-R, IM, DM	Multiple regression analysis identified significant associations between hippocampal CBF volume and GDS or DM scores in the Stage C group.
Kure et al. (20)	Cross-sectional study	36 HF 40 Healthy controls	HF 68 ± 7 Healthy 67 ± 5	59.2%	Not specified	TCD	CDRT, TMTa, TMTb, SCWT	Reduced CCA-BFV was associated with worse cognition.
Alosco et al. (21)	Cross-sectional study	60 HF/AF 127 HF/no AF	HF/AF 70.98 ± 8.59 HF/no AF 67.33 ± 8.71	69.5%	Not specified	TCD	3MS, TMTa, TMTb, DSC, FAB, CVLT-II, BNT, AFT	Partial correlations indicated significant associations between MCA-BFV with memory and language.
Alosco et al. (22)	Self-control study	65 HF	69.77 ± 10.06	72.3%	Not specified	TCD	3MS, TMTa, TMTb, FAB, CVLT-II, LDFR	Decreased baseline cerebral perfusion also emerged as a strong predictor of poorer 12-month attention/executive function.
Alosco et al. (23)	Cross-sectional study	89 HF	67.61 ± 11.78	73%	Not specified	TCD	3MS, DSC, TMTa, TMTb, LNST, FAB, CVLT-II, BNT, GPT	The global CBF-V correlated with memory performance but not with other tasks.
Alosco et al. (24)	Cross-sectional study	99 HF	67.46 ± 11.37	73.7%	Not specified	TCD	3MS, TMTb, CPT, SCWT, FAB, CVLT-II, BNT, AFT	The global lower CBF-V was associated with reduced performance on tests of attention/executive function and memory assessment.
Jesus et al. (25)	Cross-sectional study	99 HF	55 ± 12	56.6%	Not specified	TCD	MMSE	Only RMCA pulsatility index correlated with MMSE score.

*HF, heart failure; CBF, cerebral blood flow; pCASL, pseudo continuous artery spin labeling; MMSE, mini-mental state examination; MOCA, Montreal cognitive assessment; COD, carotid occlusive disease; VCI, vascular cognitive impairment; CBFV, cerebral blood flow velocity; MCA, middle cerebral artery; TCD, transcranial doppler; ARI, self-regulating index; TCD, transcranial doppler; CDRT, cognitive drug research test; CCA-BFA, common carotid arterial blood flow velocities; RMCA, right middle cerebral artery; CI, cognitive impairment; GDS, geriatric depression scale; AF, atrial fibrillation*.

### Quality Assessment of Included Studies

This systematic review included 66 relevant clinical studies, including 55 cross-sectional studies, three cohort studies, five case-control studies, and three self-control studies.

The results of using AHRQ to evaluate the risk of bias and method quality of 55 cross-sectional studies are shown in Supplementary Material 2 (Supplementary Table 1). A total of seven studies (18, 30, 37, 40, 41, 68, 82) were included with high quality, and the remaining 48 studies were with medium quality. Specifically, all the included cross-sectional studies contained clear data sources, the inclusion and exclusion criteria, and the evaluations for quality assurance purposes. No subjective factors were included that could affect each study. Only one study (67) described a possible strategy to compensate for lost data. A total of five studies (30, 63, 67, 70, 82) followed up the patients and explained the follow-up results. A total of four studies (25, 29, 32, 47) did not present a plausible explanation of how to evaluate and control confounding factors. In addition, a total of 26 studies indicated the period of identifying patients, whereas 17 studies indicated that the included subjects were continuous. A total of 23 studies explained the reasons for exclusion of patients in the analysis, and 17 studies summarized the response rate and the integrity of data collection.

The results of using NOS to evaluate the risk of bias and the method quality of five case-control studies are shown in Supplementary Material 2 (Supplementary Table 2). The mean score of NOS in the included case-control studies was 5.8. One study (27) was of high quality, and 4 studies (32, 44, 61, 66) exhibited medium quality. Specifically, all studies indicated that the diagnosis of the disease was adequate, and included the definition of control, and the determination and method of exposure. Only the most important confounding factors were controlled in the feasibility analysis. Only two studies (27, 61) collected representative cases in succession. No study indicated that the non-response rates of the study group and the control group were the same. In terms of control selection, two studies (27, 44) selected community control and three studies (32, 61, 66) hospital control, respectively.

The results of using NOS to evaluate the risk of bias and the quality of the methods of three cohort studies are shown in Supplementary Material 2 (Supplementary Table 3). The mean score of NOS in the included cohort studies was 7.3. A total of two studies (34, 35) exhibited high quality, and one study (80) was of medium quality. The exposure cohorts of all studies were well-represented, whereas the non-exposure cohorts and the exposure cohorts were from the same community. The determination of exposure and the evaluation of outcome events were reliable, and only the most important confounding factors were controlled in the feasibility analysis. In addition, two studies (34, 35) reported on optimal adequacy and completeness of the follow-up period.

The results of using MINORS to evaluate the risk of bias and the quality of the methods of three self-control studies are shown in Supplementary Material 2 (Supplementary Table 4). A total of two studies (22, 83) exhibited high quality, and one study (50) exhibited medium quality. All three studies (22, 50, 83) indicated the clarity of research purpose, the representativeness of endpoint indicators, sufficient follow-up time, and the low loss of follow-up rate. However, neither study exhibited prospective data collection, unbiased assessment of endpoint indicators, prospective calculation of the study size, and setting of a control group. In addition, only two studies (22, 83) indicated the consistency of the included patients.

### Outcome Analysis

#### Association of Brain Imaging and Cognitive Function in Patients With HF

##### Association of CBF Changes and Cognitive Function in Patients With HF

A total of eight studies reported the association between cognitive function and the changes of CBF in patients with HF. The details of the studies are shown in Table 1. Firstly, two studies (18, 20) revealed that the CBF volumes of the HF patients were significantly reduced compared with those of the control group. Secondly, seven studies (19–25) demonstrated that the decrease in CBF volume or cerebral blood flow velocity (CBF-V) was significantly associated with the impairment of multiple cognitive domains in HF patients. A longitudinal study (22) indicated that the reduced CBF volume at baseline in HF patients was a powerful predictor of poor attention/executive function following 12 months of intervention. A total of two studies demonstrated that the reduced CBF-V volumes of the common carotid arterial (CCA) (20) or the right middle cerebral artery (RMCA) (25) were significantly correlated with the decline of cognitive function in HF patients. A previous study indicated that the blood flow of the hippocampal tissues of patients with HF symptoms in stage C was significantly decreased compared with that of patients with structural heart disease without HF symptoms in stage B (19). In addition, it was positively correlated with the score of delayed memory. The changes noted in the CBF volume were examined in three studies (21, 23, 24), which elucidated that the combination of CBF decline with AF, depression, or BMI may result in higher number of adverse effects on attention, executive function, memory, and other cognitive areas of HF patients. However, one study (18) indicated that the CBF value was lower in the HF group, whereas no significant correlation was noted between CBF and CI compared with the control group.

##### Effects of MTA on Cognitive Function in Patients With HF

A total of three studies reported on the association between cognitive function and MTA in patients with HF. The details of the studies are shown in Table 2. Firstly, it was shown by two studies that MTA was more pronounced in HF patients than healthy controls (26, 27). One of these studies (27) pointed out that the degree of atrophy of the right temporal lobe was more obvious. Secondly, three studies revealed the correlation between MTA and cognitive decline in HF patients. Specifically, a previous study (26) suggested that MTA was significantly associated with the severity of overall CI and deficits in cognitive areas, such as attention and verbal memory in HF patients. Another study (28) demonstrated a similar conclusion, in which MTA was associated with memory impairment and executive dysfunction in HF patients. Furthermore, the association was found only in left MTA in a case-control study (27).

**Table 2:** The association between MTA with cognitive function in patients with HF.

**References**	**Study design**	**Participants**	**Mean age**	**Male%**	**Type of HF**	**Methods of MTA**	**Evaluation of cognitive function**	**Study results**
Frey et al. (26)	Cross-sectional study	148 HF 284 Healthy controls	HF 65 ± 11 Healthy 65 ± 10	84.5%	All types	3.0-Tesla-MRI	TAP, VVM, WMS-R, RWT, H5PT	The degree of MTA was strongly associated with the severity of CI
Beer et al. (27)	Case control study	31 HF 24 Healthy controls	HF 54.3 ± 10.6 Healthy 56.1 ± 8.2	83.6%	HFrEF EF ≤ 40%	1.5-Tesla-MRI	CAMDEX, CVLT, BVMT-R	Left medial temporal lobe atrophy showed moderate association with cognitive scores in CHF
Vogels et al. (28)	Cross-sectional study	58 HF	68.7 ± 9.1	74.1%	HFrEF, HFmrEF EF ≤ 45%	1.5-Tesla-MRI	CANTAB, TMT-B, LFT, CFT, SCWT, TMT-A, MMSE, NART	MTA was related to CI

*HF, heart failure; CHF, chronic heart failure; MTA, medial temporal lobe atrophy; HFrEF, heart failure with reduced ejection fraction; HFmrEF, heart failure with mid-range ejection fraction; HFpEF, heart failure with preserved ejection fraction; CI, cognitive impairment; All types, HFrEF, HFmrEF, HFpEF; MMSE, mini-mental state examination*.

##### Effects of the Changes Noted in GM and DM on the Cognitive Function of Patients With HF

A total of two studies reported on the association between cognitive function and the changes of GM in patients with HF. The details of these studies are shown in Table 3. Both studies (29, 30) indicated the correlation between decreased total GM and cognitive decline in HF patients. Furthermore, it was pointed out (29) that this correlation may be more evident on attention/executive function.

**Table 3:** The association between changes noted in the GM and WM with cognitive function in patients with HF.

**References**	**Study design**	**Participants**	**Mean age**	**Male%**	**Type of HF**	**Methods of GM**	**Evaluation of cognitive function**	**Study results**
Alosco et al. (29)	Cross-sectional study	81 HF	68.01 ± 8.65	60.5%	Not specified	1.5-Tesla-MRI	MMSE, FAB, DSCT, CVLT-II, BNT, AFT	Reduced total GM volume interacted with worse attention/executive function and memory to negatively impact instrumental activities of daily living
Alosco et al. (30)	Cross-sectional study	69 HF	68.07 ± 8.02	58%	Not specified	1.5-Tesla-MRI	3MS	Reduced total GM volume and decreased cortical thickness were associated with poorer 3MS scores
Frey et al. (26)	Cross-sectional study	148 HF 284 Healthy control	HF 65 ± 11 Healthy 65 ± 10	84.5%	All types	3.0-Tesla-MRI	TAP, VVM, WMS-R, RWT, H5PT	WMH seems to be not related to CI
Alosco et al. (31)	Cross-sectional study	69 HF	68.55 ± 8.07	63.8%	Not specified	1.5-Tesla-MRI	MMSE	Marginal significance between increased total WMHs and poorer performance on the MMSE
Beer et al. (27)	Cross-sectional study	31 HF 24 Healthy controls	HF 54.3 ± 10.6 Healthy 56.1 ± 8.2	83.6%	HFrEF EF ≤ 40%	1.5-Tesla-MRI	CAMDEX, CVLT, BVMT-R	Periventricular and deep WMH had fair negative correlations to total CAMCOG score.
Vogels et al. (28)	Cross-sectional study	58 HF	68.7 ± 9.1	74.1%	HFrEF, HFmrEF EF ≤ 45%	1.5-Tesla-MRI	CANTAB, TMT-B, LFT, CFT, SCWT, TMT-A, MMSE, NART	Total WMH and deep WMH were found not to correlate with cognitive measures

*HF, heart failure; HFrEF, heart failure with reduced ejection fraction; GM, gray matter; MMSE, mini-mental state examination; CHF, chronic heart failure; HFmrEF, heart failure with mid-range ejection fraction; HFpEF, heart failure with preserved ejection fraction; CI, cognitive impairment; All types, HFrEF, HFmrEF, HFpEF; WM, white matter; WMH, white matter hyperintensities; MMSE, mini-mental state examination*.

A total of four studies reported on the association between cognitive function and the changes of WM in patients with HF. The details of the studies are shown in Table 3. A total of two studies reported the moderate (27) or marginal (31) correlation between white matter hyperintensities (WMH) and cognitive function score in HF patients. However, two other studies (26, 28) revealed that WMH was not related to cognitive function scores.

#### Effects of EF on Cognitive Function in Patients With HF

The correlation between EF and cognitive function in patients with HF was reported in nine studies, as well as in seven studies with differences in cognitive function among different subtypes of HF. The details of the studies are shown in Table 4.

**Table 4:** The association between EF and cognitive function in patients with HF.

**References**	**Study design**	**Participants**	**Mean age**	**Male %**	**Characteristics of objects with HF**	**Evaluation of cognitive function**	**Study results**
D'Elia et al. (32)	Case controlled design	12 No HF 12 HFpEF 12 HFrEF	No HF 69.3 HFpEF 76 HFrEF 71.1	61.1%	NYHA classes ≥ III	TMT-A, TMT-B, DSF, DSB, LM, SSF, SSB, ACE-R	Compared to HFrEF patients and controls, HFpEF patients showed lower cognitive scores at baseline tests
Faulkner et al. (33)	Cross-sectional study	5,268 No HF 205 HFpEF 60 HFrEF	No HF 75.4 ± 5.1 HFpEF 77.2 ± 5.6 HFrEF 77.6 ± 5.4	58.4%	(LVEF) HfpEF 62.7 ± 6.3% HFrEF 39.9 ± 6.7%	DWR, LMTa, IL, LMTb, DSB, DSST, TMT-A, TMT-B, AN, BNT, WF, MMSE	Neurocognitive test scores were not significantly different among participants with HfpEF and HFrEF
Hammond et al. (34)	cohort study	496 HF 4,368 No HF	HF 75.7 ± 6.0 No HF 73.7 ± 5.3	41.6%	Not specified	3MSE, DSST	After HF diagnosis, rates of cognitive decline by EF category were not significantly different
Witt et al. (35)	cohort study	953 HF 5,542 No HF	HF 76.1 ± 5.2 No HF 76.3 ± 5.3	41.1%	Not specified	DWR, DSST, WF	Those with HFrEF showed similar cognitive test results to those with HFpEF
Warraich et al. (36)	Cross-sectional study	96 HFpEF 106 HFrEF	HFpEF 71.7 ± 7.4 HFrEF 72.3 ± 7.7	46.0%	(LVEF) HFpEF 55% HFrEF 28% NYHA classes II-IV	MOCA	CI based on MOCA score was similar between HFpEF and HFrEF
Adebayo et al. (37)	Cross-sectional study	26 HFpEF 14 HFrEF	HFpEF 65.11 ± 11.43 HFrEF 57.57 ± 14.88	62.5%	(LVEF) HFpEF 37.54% HFrEF 47.00% NYHA classes I-IV	CSI'D, WLLD, BNT, MTT	Compared with HFrEF patients, the scores were lower in the CSI'D and WLLDR in HFpEF patients
Shin et al. (38)	Cross-sectional study	82 HF	65.13 ± 9.80	59.8%	LVEF < 52% for men and < 54% for women (Abnormal LV systolic function) NYHA classes I-IV	SVLT, DST, COWA, KTMT	The attention of patients with severe systolic abnormalities (LVEF < 30%) was significantly lower than that of patients with mild to moderate systolic abnormalities (LVEF ≥ 30%)
Albabtain et al. (39)	Cross-sectional study	90 HFpEF 31 HFrEF	66 ± 10	91%	(LVEF) HFpEF 55 ± 6% HFrEF 25 ± 8% NYHA classes I-IV	MOCA	Patients with HFrEF had lower MoCA scores as compared to HFpEF patients
Graham et al. (40)	Cross-sectional study	2,043 HF and without prior stroke	60.8 ± 11.3	80%	LVEF 24.6% NYHA classes I-IV	MMSE	LVEF was not associated with the MMSE
Feola et al. (41)	Cross-sectional study	303 HF	71.6 ± 9.9	59.4%	LVEF 43.4 ± 15.8% NYHA classes I-IV	MMSE	A significant correlation not between MMSE and LVEF was observed
Steinberg et al. (42)	Cross-sectional study	55 HF	55.3 ± 7.8	80.0%	LVEF 22.4 ± 12.8% NYHA classes I-III	TMT-A, VLL, DST, MMSE, CIS	LVEF as being significantly associated with subjective CI
Festa et al. (43)	Cross-sectional study	207 HF	17 - 72	74%	Not specified	HVLT, BVMT, WAIS III, TMT-A, TMT-B, COWA	Patients 63 years or older showed a significant decline in memory performance when EF dropped below 30%
Sauvé et al. (44)	Case control study	50 HF 50 Healthy controls	HF 63 ± 14 Healthy 62.5 ± 14	49%	LVEF 27 ± 14% NYHA classes II-IV	SDMT, RAVLT, COWA, FTT, MST, VST, WAIS	CI severity was not associated with LVEF
Hoth et al. (45)	Cross-sectional study	31 HF 31 No HF	HF 69.1 ± 8.5 No HF 68.9 ± 8.5	49.5%	LVEF 31.5 ± 9.1% NYHA classes II-IV	TMT-A, TMT-B, COWA, WAIS III, SCWT	EF was associated with weaker global cognition, performance on attention, working memory, and fluency
Feola et al. (46)	Cross-sectional study	60 HF	65.5	73.3%	LVEF 32.9% NYHA classes II-IV	MMSE, CBT, VST, PMT	A significant correlation not between MMSE and LVEF was observed
Zuccalà et al. (47)	Cross-sectional study	57 HF	77 ± 1	28%	LVEF 44.7 ± 2.3% NYHA classes II-III	MMSE, MDB	The MMSE score was associated with LVEF according to a non-linear correlation, so that cognitive performance was significantly lower in subjects with LVEF ≤ 30%

*HF, heart failure; CHF, chronic heart failure; HFrEF, heart failure with reduced ejection fraction; HFmrEF, heart failure with mid-range ejection fraction; HFpEF, heart failure with preserved ejection fraction; CI, cognitive impairment; EF, ejection fraction; LVEF, left ventricular ejection fraction; MMSE, mini-mental state examination; MOCA, Montreal cognitive assessment*.

Previous studies (four in total) that examined the association between EF and cognitive function (40, 41, 44, 46) suggested no significant correlation between these two parameters. However, a set of five different studies exhibited different results. These data indicated that EF was significantly correlated with cognition in HF, notably when it was <30%, which was noted in the total cognitive ability (45, 47), attention (38), memory (43), and subjective cognition (42), respectively.

A total of seven (32–37, 39) studies investigated the differences in cognitive function among patients with different types of HF. Among them, two large prospective cohort studies (34, 35) (*n* = 4,864/6,495) indicated no significant differences in the decline of cognitive function scores in heart failure with reduced ejection fraction (HFrEF) and heart failure with preserved ejection fraction (HFpEF) patients following 5 or 15 years of follow-up, respectively. Similar results were obtained from the other two observational studies (33, 36). However, three studies had reached opposite conclusions, and two studies (32, 37) pointed out that patients with HFpEF exhibited lower cognitive function scores at baseline compared with those of patients with HFrEF and control subjects. Finally, it was also shown (39) that HFrEF exhibited lower MOCA scores relative to HFpEF.

#### Effects of Specific Detection Indices on Cognitive Function in Patients With HF

##### Effects of BMI on Cognitive Function in Patients With HF

A total of seven studies reported the association between BMI and cognitive function in patients with HF. The details of studies are shown in Table 5. A total of two studies (37, 49) reported that the increase in BMI was significantly correlated with the decrease in the cognitive function of HF patients. BMI was associated with multiple cognitive domains in HF patients including attention, language, and executive function (24, 50, 51). Furthermore, for every 1 unit decrease in BMI, the improvement of the executive function in patients with HF was 1.4% (50). However, another two studies (40, 48) exhibited the opposite conclusions, and it was shown that negative correlations were present between BMI and cognitive function in HF patients.

**Table 5:** The association between BMI and cognitive function in patients with HF.

**References**	**Study design**	**Participants**	**Mean age**	**Male %**	**Type of HF**	**Evaluation of cognitive function**	**Detection results**
Ely et al. (48)	Cross-sectional study	117 HF	72.6 ± 11.46	49%	All types	Mini-Cog	At lower EF%, and with higher anxiety, patients without obesity might be at greater risk of CI than those with obesity
Adebayo et al. (37)	Cross-sectional study	26 HFpEF 14 HFrEF	HFpEF 65.11 ± 11.43 HFrEF 57.57 ± 14.88	62.5%	All types	CSI'D, WLLD, BNT, MTT	BMI showed an inverse relationship with the total CSI'D score in the cohort with HFrEF
Alosco et al. (49)	Cross-sectional study	200 HF	68.19 ± 9.00	64%	Not specified	TMT-A, TMT-B, CVLT-II, AFT, BNT	BMI emerged as a significant predictor of cognitive function, with higher BMI predictive of worse CI
Graham et al. (40)	Cross-sectional study	2,043 HF	60.8 ± 11.3	80%	HFrEF	MMSE	BMI was independently positively associated with the MMSE in HF patients
Alosco et al. (50)	Self-control study	50 HF	66.7 ± 7.9	74%	Not specified	NTB	On average, for every 1 unit decrease in BMI, the improvement of executive function in patients with HF was 1.4%
Hawkins et al. (51)	Cross-sectional study	231 HF	68.7 ± 7.3	66%	Not specified	TMT-A, TMT-B, SCWT, LNST, RAVLT, RCFLD, 3MSE	For men, greater BMI predicted poorer attention and executive function. In women, greater BMI was not associated with any cognitive variable
Alosco et al. (24)	Cross-sectional study	99 HF	67.46 ± 11.37	73.7%	Not specified	3MSE, TMT-B, CPT, SCWT, FAB, CVLT-II, BNT, AFT	Elevated BMI was independently associated with reduced attention/executive function and language test performance

*HF, heart failure; CHF, chronic heart failure; HFrEF, heart failure with reduced ejection fraction; HFmrEF, heart failure with mid-range ejection fraction; HFpEF, heart failure with preserved ejection fraction; CI, cognitive impairment; EF, ejection fraction; MMSE, mini-mental state examination; BMI, body mass index*.

##### Effects of Electrolyte Levels on Cognitive Function in HF Patients

A total of five studies reported on the correlation between electrolyte levels and cognitive function in patients with HF. The details of the studies are shown in Table 6. A large secondary analysis study (55) (*n* = 1,511) indicated that serum sodium (<135 mEq/l) and potassium (<3.6 mEq/l) levels exhibited a significant negative correlation with the degree of CI in patients with HF. An additional study (39) suggested that hypokalemia significantly increased CI, whereas no correlation was found. In addition, high sodium levels may also exhibit adverse effects on cognitive function in patients with HF, and a pertinent correlation was noted in a study (54) following completion of a dietary questionnaire. In contrast to these findings, a previous study (53) demonstrated that in patients with HF who ingested excessive sodium and insufficient potassium, the increased Na/K ratio was significantly associated with CI. However, in another study (52), a different conclusion was reached, and it was suggested that by measuring urinary sodium excretion in HF patients, sodium consumption exceeded the recommended amounts, and was unrelated to cognitive function.

**Table 6:** The association between electrolyte and cognitive function in patients with HF.

**References**	**Study design**	**Participants**	**Mean age**	**Male %**	**Type of HF**	**Evaluation of cognitive function**	**Test samples**	**Detection results**
Albabtain et al. (39)	Cross-sectional study	121 HF	66 ± 10	91%	All types	MOCA	Serum sodium	No significant correlation was found between sodium levels and MoCA score
Dolansky et al. (52)	Cross-sectional study	159 HF without two urine samples 180 HF with two urine samples	HF without two urine samples 69.14 ± 10.29 HF with two urine samples 68.72 ± 8.92	61.1%	HFrEF	3MS, SCWT, TMT-A, TMT-B, LNS, SCWT, FAB, RAVLT, TH, SD; LDS	Urine sodium	Adherence to urine sodium collection was poor, especially among those with poorer cognitive function. Sodium consumption was unrelated to cognitive function
Hwang et al. (53)	Cross-sectional study	91 HF	57.0 ± 14.1	67.0%	All types	K-MMSE, SVLT, COWAT	Dietary sodium and potassium intake	Elevated sodium-to-potassium ratios showed significant negative associations with cognitive function (memory) in HF patients
Alosco et al. (54)	Cross-sectional study	152 HF	67.59 ± 10.79	60.5%	Not specified	3MS, TMT-A, TMT-B, DSC, SCWT, LNS, CVLT-II, BNT, ANT	Dietary Habits	The questionnaire showed that revealed consumption of foods high in sodium was associated with reduced cognitive function
Zuccalà et al. (55)	Cross-sectional study	1,511 HF	CI 82 ± 8 No CI 76 ± 10	29.71%	Not specified	Hodkinson AMT	Serum sodium and potassium	Sodium and potassium levels were independently associated with CI

*HF, heart failure; CHF, chronic heart failure; HFrEF, heart failure with reduced ejection fraction; HFmrEF, heart failure with mid-range ejection fraction; HFpEF, heart failure with preserved ejection fraction; CI, cognitive impairment; EF, ejection fraction; MOCA, Montreal cognitive assessment*.

##### Effects of BNP/NT-proBNP Levels on Cognitive Function in HF Patients

A total of six studies reported on the association between cognitive function and BNP/NT-proBNP levels in patients with HF. The details of these studies are shown in Table 7. A large cross-sectional study (82) discovered (*n* = 951) that BNP was an indicator of severity in HF patients, and exhibited a significant correlation with the MMSE score. Similar results have been shown by other studies (46, 47). Another study (57) indicated a correlation between Log BNP and ACE-R (Addenbrooke's cognitive examination-revised) fluency subtest in HF patients. In addition, NT-proBNP was independently associated with cognitive function in patients with HF (81). Notably, van Vliet et al. (83) described that the combination of high NT-proBNP levels and low systemic BP could predict the decline in cognitive function in HF patients following 5 years of follow up and adjustment for the relevant confounding factors.

**Table 7:** The association between BNP/NT-proBNP and cognitive function in patients with HF.

**References**	**Study design**	**Participants**	**Mean age**	**Male %**	**Type of HF**	**Evaluation of cognitive function**	**Detection results**
Pierobon et al. (57)	Cross-sectional study	100 HF	74.9 ± 7.1	74%	Not specified	MMSE, ACE-R, FAB, PFT, CDT, TMT-A, TMT-B	Log-BNP was negatively correlated to ACE-R-Fluency subtest
Dong et al. (81)	Cross-sectional study	96 HF	CI 64.3 ± 8.3 No CI 54.2 ± 10.1	85.4%	All types	MMSE, MOCA	NT-proBNP was positively and independently associated with CI
Leto et al. (82)	Cross-sectional study	951 HF	70.8 ± 10.3	63%	All types	MMSE	An negative correlation between BNP and MMSE scores at admission and discharge emerged
van Vliet et al. (83)	Self-control study	560 HF	≥ 85	22.7%	Not specified	MMSE	In the oldest old, high NT-proBNP levels were associated with lower MMSE scores
Feola et al. (41)	Cross-sectional study	303 HF	71.6 ± 9.9	59.4%	All types	MMSE	At multivariate analysis only MMSE and BNP were inversely correlated significantly
Feola et al. (46)	Cross-sectional study	60 HF	65.5	73.3%	All types	MMSE, CBTT, VST, PMT	A negative correlation between MMSE and BNP was observed

*HF, heart failure; CHF, chronic heart failure; HFrEF, heart failure with reduced ejection fraction; HFmrEF, heart failure with mid-range ejection fraction; HFpEF, heart failure with preserved ejection fraction; CI, cognitive impairment; MMSE, mini-mental state examination; BNP, B-type natriuretic peptide; NT-proBNP, N-terminal pro-B-type natriuretic peptide*.

#### Effects of Comorbidities on Cognitive Function in Patients With HF

##### Effects of Depression on Cognitive Function in Patients With HF

A total of 15 studies reported on the association between cognitive function and depression in patients with HF. The details of these studies are shown in Table 8. A total of 11 studies demonstrated a correlation between the aforementioned parameters. A total of six studies (56, 57, 59, 61, 63, 66) reported that depression was a significant risk factor for cognitive decline in HF patients. Furthermore, the association was mainly driven by non-somatic symptoms of depression (60). Depression can affect multiple cognitive fields in HF patients. A total of three studies (23, 62, 64) indicated that depression was related to the impairment of memory function, attention, executive ability, language ability, and other cognitive fields in HF patients. In addition, the combined effects of depression and physical weakness increased the risk of CI in HF patients (58). However, four studies had found opposite results. A total of three cross-sectional studies (42, 48, 65) suggested that no significant correlation was present between depressive symptoms and/or the severity of depression and cognitive function in HF patients. Similar results were obtained from another case-control study (44).

**Table 8:** The association between depression and cognitive function in patients with HF.

**References**	**Study design**	**Participants**	**Mean age**	**Male %**	**Type of heart failure**	**Evaluation of cognitive function**	**Evaluation of depression**	**Detection results**
Sargent et al. (56)	Cross-sectional study	113 HF	NYHA II 57.74 ± 1.42 NYHA III–IV 55.66 ± 1.74	65%	Not specified	MOS, 6-item Likert scale	HADS	Depression was significantly negatively correlated with cognitive function in HF patients
Pierobon et al. (57)	Cross-sectional study	100 HF	74.9 ± 7.1	74%	Not specified	MMSE, ACE-R, FAB, PFT, CDT, TMT-A, TMT-B	GDS	ACE-R total and CDT adjusted scores both have a negative relation with depression scores
Ely et al. (48)	Cross-sectional study	117 HF	72.6 ± 11.46	49%	All types	Mini-Cog	PHQ-9	Depression was not significantly related to cognitive function
Lee et al. (58)	Cross-sectional study	289 HF	62.84 ± 11.81	78.5%	All types	MMSE-K	PHQ-9, DSM-IV	The combined influence of depression and physical frailty increased the risk of CI
Taraghi et al. (59)	Cross-sectional study	184 HF	Women 70.7 ± 8.35 Men 70.01 ± 8.99	38.6%	All types	Iranian version of AMT	GDS	There were significantly negative relationships between cognitive status and geriatric depression scale
Hawkins et al. (60)	Cross-sectional study	326 HF	68.6 ± 9.7	59.5%	Not specified	SCWT, TMT-A, TMT-B, LNST, FAB, RAVLT, TH, SD, LDS	PHQ-9	Greater overall depressive symptom severity was associated with poorer performance on multiple cognitive domains, an effect driven primarily by the non-somatic symptoms of depression
Hjelm et al. (61)	Case control study	138 HF 564 No HF	HF 86.56 ± 3.69 No HF 85.22 ± 2.85	66.7%	Not specified	MMSE, CDT	Not specified	Depression, was associated with a higher risk for dementia in individuals with CHF
Hanon et al. (62)	Cross-sectional study	912 HF	79.1 ± 5.8	65%	All types	MIS-D	Not specified	Depression was independent determinants of memory impairment
Pulignano et al. (63)	Cross-sectional study	190 HF	77 ± 5.1	53.7%	All types	MMSE, WDS	GDS	Depressive symptoms were significantly negative associated with CI
Alosco et al. (23)	Cross-sectional study	89 HF	67.61 ± 11.78	73%	Not specified	3MS, DS, TMT-A, TMT-B, LNS, FAB, CVLT-II, BNT, GPT	BDI-II	Depression was associated with reduced performance on tasks assessing attention/executive function and motor function
Garcia et al. (64)	Cross-sectional study	116 HF	68.53 ± 9.30	63.5%	Not specified	3MS, TMT-A, TMT-B, FAB, DS, CVLT, CFT, BNT, AN, GPT	BDI-II	Greater depressive symptoms were associated with poorer performance on tests of attention, executive function, psychomotor speed, and language
Steinberg et al. (42)	Cross-sectional study	55 HF	55.3 ± 7.8	80.0%	HFrEF, HFmrEF	TMT-A, VLL, DST, MMSE, CIS	SCID, HADS	No significant association was found between depressive symptoms and CI
Sauvé et al. (44)	Case control study	50 HF 50 Healthy controls	HF 63 ± 14 Healthy 62.5 ± 14	49%	HFrEF	SDMT, RAVLT, COWA, FTT, MST, VST, WAIS	MHI	CI severity was not associated with depression
Akomolafe et al. (65)	Cross-sectional study	100 HF	67.5 ± 9.01	36%	Not specified	MMSE	GDS	There was no significant relationship between CI and depression of CHF
Trojano et al. (66)	Case-control study	149 HFm 159 HFs 207 No HF	HFm 74.7 ± 7.1 HFs 76.8 ± 7.3 No HF 73.7 ± 6.6	46%	Not specified	AM, RCPM, CVFT, CBTT, VWS, RALT, MMSE	GDS	The following qualified as independent correlates of the outcome at logistic regression analysis: depression

*HF, heart failure; CHF, chronic heart failure; HFrEF, heart failure with reduced ejection fraction; HFmrEF, heart failure with mid-range ejection fraction; HFpEF, heart failure with preserved ejection fraction; CI, cognitive impairment; HADS, hospital anxiety and depression scale; MMSE, mini-mental state examination; GDS, geriatric depression scale; PHQ-9, patient health questionnaire-9; DSM-IV, diagnostic and statistical manual of mental disorders, 4th edition; HFm, patients with moderate HF (NYHA Class II); HFs, patients with severe HF (NYHA Classes III and IV)*.

##### Effects of AF on Cognitive Function in Patients With HF

A total of five studies reported on the association between cognitive function and AF in patients with HF. The details of the studies are shown in Table 9. A large cross-sectional study (69) (*n* = 881) suggested that permanent atrial fibrillation (permAF) was an independent risk factor for CI in HF patients. The same conclusion was reported in another cohort study (70) (*n* = 331). A total of two cross-sectional studies (21, 68) further showed that HF patients with AF exhibited worse cognitive function and memory ability compared with patients without AF. In addition, in terms of cognitive function, HF and AF interacted with each other. In a large prospective cross-sectional study (67) (*n* = 1,244) with 1-year follow-up, it was shown that HF was independently associated with CI at baseline in AF patients, but not with cognitive function after follow-up.

**Table 9:** The association between AF and cognitive function in patients with HF.

**References**	**Study design**	**Participants**	**Mean age**	**Male %**	**Type of HF**	**Evaluation of cognitive function**	**Detection results**
Wang et al. (67)	Cross-sectional study	463 HF/AF 781 no HF/ AF	HF/AF 76.9 ± 7.3 no HF/AF 74.7 ± 6.9	51.2%	Not specified	MOCA	HF was independently associated with baseline CI, but not with developing CI at 1 year
Yang et al. (68)	Cross-sectional study	72 HF/AF 116 HF/no AF	HF/AF 68.9 ± 9.6 HF/no AF 64.6 ± 10.9	62.8%	Not specified	MOCA	In HF patients, AF was associated with poorer performance on cognitive function
Coma et al. (69)	Cross-sectional study	187 HF/AF 694 HF/no AF	HF/AF 75.9 ± 8.5 HF/no AF 71.7 ± 11.5	56%	All types	MMSE, SPSMQ	The presence of permAF is positively associated with CI in patients with HF, both with HFrEF and HFpEF
Pulignano et al. (70)	Cross-sectional study	98 HF/AF 233 HF/no AF	HF/AF 78 ± 5.5 HF/no AF 77 ± 5.3	57.7%	All types	cMMSE	At multivariable analyses, AF emerged as positively related to CI
Alosco et al. (21)	Cross-sectional study	60 HF/AF 127 HF/no AF	HF/AF 70.98 ± 8.59 HF/no AF 67.33 ± 8.71	69.5%	Not specified	3MS, TMT-A, TMT-B, DSC, FAB, CVLT-II, BNT, AFT	The association between AF and 3MS remained significant

*HF, heart failure; CHF, chronic heart failure; HFrEF, heart failure with reduced ejection fraction; HFmrEF, heart failure with mid-range ejection fraction; HFpEF, heart failure with preserved ejection fraction; CI, cognitive impairment; AF, atrial fibrillation; MOCA, Montreal cognitive assessment; MMSE, mini-mental state examination*.

##### Effects of DM on Cognitive Function in Patients With HF

A total of six studies reported on the association between cognitive function and diabetes mellitus (DM) in patients with HF. The details of the studies are shown in Table 10. A total of two studies (55, 69) indicated that diabetes was independently associated with CI in patients with HF following adjustment for potential confounding factors, while three studies (49, 71, 72) demonstrated that DM was positively correlated with cognitive dysfunction. Another large longitudinal study (61) (*n* = 702) further suggested that DM was particularly related to an increased risk of 10-year progression to vascular dementia in HF patients.

**Table 10:** The association between DM and cognitive function in patients with HF.

**References**	**Study design**	**Participants**	**Mean age**	**Male %**	**Type of HF**	**Evaluation of cognitive function**	**Detection results**
Coma et al. (69)	Cross-sectional study	881 HF	72.6 ± 11.1	56.4%	All types	MMSE, SPSMQ	In the multivariate analysis, CI was positively associated with DM
Alosco et al. (49)	Cross-sectional study	200 HF	68.19 ± 9.00	64%	Not specified	TMT-A, TMT-B, CVLT-II, AFT, BNT	A diagnostic history of T2DM was negatively associated with decreased cognitive function
Hjelm et al. (61)	Case control study	138 HF 564 No HF	HF 86.56 ± 3.69 No HF 85.22 ± 2.85	66.7%	Not specified	MMSE, CDT	DM was specifically associated with an increased risk for vascular dementia
Basile et al. (71)	Cross-sectional study	43 HF/DM 36 HF/no DM	HF/DM 78.9 ± 7.9 HF/no DM 77.6 ± 8	50.6%	All types	MMSE	From univariate analysis, a MMSE score < 24 was found to be directly correlated with DM
Alosco et al. (72)	Cross-sectional study	59 HF/DM 110 HF/no DM	HF/DM 68.05 ± 8.42 HF/no DM 68.85 ± 11.18	65.7%	Not specified	3MS, TMT-A, TMT-B, DSC, LNS, CVLT-II, BNT, AFT, GPT	HF patients with T2DM evidenced significantly greater impairments across multiple cognitive domains than HF patients without T2DM
Zuccalà et al. (55)	Cross-sectional study	1,511 HF	CI 82 ± 8 No CI 76 ± 10	29.71%	Not specified	Hodkinson AMT	Hyperglycemia was positively associated with CI

*HF, heart failure; CHF, chronic heart failure; HFrEF, heart failure with reduced ejection fraction; HFmrEF, heart failure with mid-range ejection fraction; HFpEF, heart failure with preserved ejection fraction; CI, cognitive impairment; T2DM, type 2 diabetes mellitus; MMSE, mini-mental state examination*.

##### Effects of Anemia on Cognitive Function in Patients With HF

A total of six studies reported on the association between cognitive function and anemia in patients with HF. The details of the studies are shown in Table 11. Multiple studies (40, 55, 59, 63, 71, 73) elucidated that the severity of anemia was negatively associated with cognitive status in patients with HF. Furthermore, three studies (40, 55, 63) indicated that the correlation existed independently. Compared with non-anemic HF patients, the combined anemia increased the risk of CI (73).

**Table 11:** The association between anemia and cognitive function in patients with HF.

**References**	**Study design**	**Participants**	**Mean age**	**Male %**	**Type of HF**	**Evaluation of cognitive function**	**Detection results**
Kim et al. (73)	Cross-sectional study	64 HF/anemia 117 HF/no anemia	70.01 ± 7.62	75.1%	All types	3MS	The multiple logistic regression indicated that compared to a non-anemic status, anemia increased the risk of CI
Taraghi et al. (59)	Cross-sectional study	184 HF	Men 70.01 ± 8.99 Women 70.7 ± 8.35	38.6%	All types	Iranian version of the AMT	There was positively significant relationship between CI and anemia
Pulignano et al. (63)	Cross-sectional study	81 HF/anemia 109 HF/no anemia	77 ± 5.1	53.7%	All types	MMSE, WDS	Anemia was positively associated with CI
Graham et al. (40)	Cross-sectional study	2,043 HF and without prior stroke	60.8 ± 11.3	80%	HFrEF	MMSE	There was an independent positive correlation between hemoglobin level and MMSE
Basile et al. (71)	Cross-sectional study	79 HF	78.3 ± 7.6	50.6%	All types	MMSE	From univariate analysis, a MMSE score was found to be directly correlated with anemia (Hb ≤ 11.9 mg/dL)
Zuccalà et al. (55)	Cross-sectional study	1,151 HF	CI 82 ± 8 No CI 76 ± 10	29.71%	Not specified	Hodkinson AMT	Anemia was positively associated with CI, after adjusting for potential confounders

*HF, heart failure; CHF, chronic heart failure; HFrEF, heart failure with reduced ejection fraction; HFmrEF, heart failure with mid-range ejection fraction; HFpEF, heart failure with preserved ejection fraction; CI, cognitive impairment; MMSE, mini-mental state examination; AMT, Abbreviated mental test; Hb, hemoglobin*.

##### Effects of Sleep Disorder on Cognitive Function in Patients With HF

A total of eight studies reported on the association between cognitive function and sleep disorders in patients with HF. The details of the studies are shown in Table 12. The effects of sleep on cognitive function in HF patients are contradictory. However, the majority of the large-sample studies have deduced that sleep will affect cognitive function. Daytime sleepiness is closely related to CI in HF patients, which would lead to the decline of memory, executive function (77), and attention (78). HF patients were accompanied by excessive daytime sleepiness (EDS) and were more likely to exhibit problems of failure to adhere to medication, which may lead to a decline in attention function (80). Furthermore, insomnia was associated with the decline of overall cognitive function in HF patients (79). Another cross-sectional study (76) further revealed that cognitive function in HF patients of different ages was related to different types of sleep disorders. Specifically, cognitive function was associated with daytime sleepiness in adults and poor nighttime sleep quality in older adults. However, this study (75) did not indicate a correlation between daytime sleepiness and cognitive function in HF patients. In addition, sleep-related breathing disorders may also affect cognitive function in HF patients. A significant negative correlation was noted between cognitive function score and oxygen saturation index (ODI) (32) in HF patients compared with the apnea-hypopnea index (AHI) (74).

**Table 12:** The association between sleep disorders and cognitive function in patients with HF.

**References**	**Study design**	**Participants**	**Mean age**	**Male %**	**Type of HF**	**Evaluation of cognitive function**	**Evaluation of Sleep quality**	**Detection results**
D'Elia et al. (32)	Case controlled design	12 HFrEF 12 HFpEF 12 No HF	HFrEF 71.1 HFpEF 76 No HF 69.3	61.1%	All types	TMT-A, TMT-B, DST, DSB, LM, SSF, SSB, ACE-R	Polysomnography	In both HFrEF and HFpEF, a significant negative correlation between ACE-R and ODI was observed
Moon et al. (74)	Cross-sectional study	28 HF	67.93 ± 5.78	40%	All types	WAIS-R, TMT-A, TMT-B, RAVLT	Sleep-monitoring device	Cognitive scores was not associated with the AHI
Walter et al. (75)	Cross-sectional study	267 HF	69.09 ± 9.32	59.6%	Not specified	MMSE, TMT-A, LNS, SCWT, RAVLT, TMT-B	PSQI	Cognitive function in HF is not associated with sleep quality or daytime sleepiness
Byun et al. (76)	Cross-sectional study	105 Adults HF 167 Elders HF	Adults HF 50.0 ± 7.74 Elders HF 69.8 ± 7.42	64.3%	Not specified	PVT, TMT-B, DSST, PMRT, LNS	ESS, PSQI, AHI	In adults, daytime sleepiness was associated with CI, whereas poor nighttime sleep quality was associated with CI in elders
Moon et al. (77)	Cross-sectional study	97 HF 744 No HF	97 HF 84.80 ± 7.62 744 No HF 81.26 ± 6.93	41.0%	Not specified	DST, WLL, WR, WMS, LM, DR, COWAT	NPI	Only daytime sleepiness mediated the relationship between the presence of HF and cognitive domains after controlling for covariates
Moon et al. (78)	Cross-sectional study	68 HF	72.32 ± 11.39	63%	Not specified	DST, CT, LL, SMT, TMT-B, LFT	PSQI, ESS	Neither sleep quality or excessive daytime sleepiness were related to cognitive function, but daytime dysfunction was related to lower letter fluency and attention index
Hjelm et al. (79)	Cross-sectional study	137 HF	With ApneaLink 71 (63–78) Without ApneaLink 77 (65–83)	68%	Not specified	MMSE, TMT-A, TMT-B, WKT, ROCF, BDT	ESS, MISS, AHI	Insomnia was associated with decreased global cognition
Riegel et al. (80)	Cohort study	280 HF	61.9 ± 12.5	64.3%	Not specified	PVT, TMT-B, DSST, PMRT, LNS, ANART	ESS	Adults with HF and EDS are more likely to have problems adhering to their medication regimen than those without EDS, the only cognition measure significantly associated with medication adherence was attention

*HF, heart failure; CHF, chronic heart failure; HFrEF, heart failure with reduced ejection fraction; HFmrEF, heart failure with mid-range ejection fraction; HFpEF, heart failure with preserved ejection fraction; CI, cognitive impairment; MMSE, mini-mental state examination; PSQI, Pittsburgh sleep quality index; ESS, epworth sleepiness scale; AHI, anea hypopnea index; NPI, psychiatric Inventory; MISS, minimal insomnia symptom scale; SDB, sleep-disordered breathing; ODI, oxygen desaturation index; ApneaLink, a portable two-channel screening tool; EDS, excessive daytime sleepiness*.

## Discussion

CI is a common concurrent disease of HF, with a high incidence in the clinic. It exerts negative effects on the prognosis of HF patients. Although the etiology and pathological mechanism of this disease are not completely clear, it is undeniable that this is due to the interaction and common effects of multiple factors. An increasing number of studies have explored the discovery of the risk factors and imaging changes of CI in patients with HF. The risk factors affecting cognitive function in HF patients include decreased EF and electrolyte levels, notably sodium and potassium ions, or proportion imbalance, high BMI, increased BNP, and co-morbidities such as AF, DM, anemia, depression, and sleep disorders. The data indicated that these risk factors exhibited negative effects on cognitive function by different mechanisms of action and that they could affect the prognosis of HF patients.

### The Effects of Imaging Changes on Cognitive Function in Patients With HF

HF is caused by cardiac filling and/or systolic dysfunction as a result of various factors, which leads to a decrease in cardiac output and cannot meet the needs of tissue metabolism. Brain damage caused by reduced cardiac output may be an important cause of CI in patients with HF and lead to related brain imaging changes. The related imaging changes mainly include the CBF, medial temporal lobe, WM, GM, and hippocampus. At present, the main findings reported on the changes of the hippocampus in HF patients are that the volumes of the hippocampus and parahippocampal gyrus are smaller (84–86), whereas only one study (86) reported on the correlation between cognitive function and hippocampal changes. Therefore, the present study only systematically analyzed the impact of the other three imaging changes on the cognitive function of HF patients.

In patients with HF, low cardiac output or impaired automatic regulation of vascular tension may lead to decreased CBF and hypoxia in the brain tissue (87, 88). The brain is very sensitive to ischemia and hypoxia, which may lead to neuron loss, glial cell proliferation, and activation of oxidative stress (20, 89). These often occur in the prefrontal lobe, temporal lobe, hippocampus, amygdala, and other parts, which may result in the decline of cognitive function (90).

In the current systematic review, seven studies (19–25) demonstrated the correlation between the decline of CBF and CI in HF patients with the exception of one study (18). The possible reasons for the negative result are as follows: Firstly, the present study did not control the confounding factors, such as hypertension and diabetes. The proportion of these HF patients was higher than those complicated with these diseases in the control group. Moreover, the study included all types of HF patients and did not analyze the correlation between cognitive function and CBF of different types of HF. It is important to note that the present study used large ROI (frontal, parietal, temporal, and occipital brain lobes), which may mask the subtle regional associations with cognitive function. Moreover, previous studies (21, 23, 24) have found that the combination of CBF decline with AF, depression, and BMI may further damage the cognitive function of HF patients, possibly due to the fact that AF, BMI, and depression can also aggravate the CI of HF patients or affect cerebral hemodynamics. Furthermore, the different measures of CBF used in the included studies affected the correlation results. A total of two studies (18, 19) used MRI to detect the CBF of participants, while other studies (20–25) used TCD to detect the blood flow velocity of cerebral vessels to represent the changes of CBF. In contrast to these observations, the use of MRI can objectively reflect the changes of CBF in HF patients, since TCD is more likely to be affected by the examiner's technique. In conclusion, the decrease in CBF volume may be associated with CI in HF patients.

The temporal lobe is located below the lateral fissure of the brain and the main functions are related to hearing, language, and memory. The medial temporal lobe mainly includes the hippocampus and its surrounding areas, which play an important role in the generation and storage of memory. The decrease of EF in HF patients leads to cerebral ischemia and hypoxia, which may lead to MTA. A previous study (91) has shown that compared with the healthy control group, the MTA of HF patients was significantly increased. MTA may lead to a series of memory problems. Several studies (92, 93) suggested that MTA was related to CI caused by various factors, including Alzheimer's and cerebrovascular diseases. Furthermore, the degree of MTA could be used as a clinical marker for the transformation from mild CI to dementia (94).

In the present system review, three studies (26–28) had reached similar conclusions and demonstrated a correlation between MTA and cognitive decline in patients with HF. It is interesting to note that CI in patients with HF was only associated with left MTA, but not with right MTA as demonstrated by a previous study (27). The possible explanation for this finding was that the observed differences may be false and that they could be attributed to methodological factors, small sample size (*n* = 55) as well as the relatively young age of the included HF patients.

GM and WM are important components of the central nervous system. GM is mainly composed of neuronal cell bodies, which is the part of the central nervous system that deeply processes a variety of information, such as sensation and movement. WM is mainly composed of projections surrounded by myelin sheath in neurons, which mainly play a role in the transmission of information. Brain hypoxia/ischemia in HF patients caused due to long-term decline of cardiac output may lead to chronic damage in brain tissues, such as GM and WM (95, 96). In HFpEF patients, diastolic dysfunction may lead to systemic hypoperfusion as a result of the reduction of left ventricular flow, resulting in the development of WM lesions (97). In HF patients, loss of GM may occur to a higher extent in the bilateral parahippocampal gyrus, bilateral cingulate gyrus, left superior temporal gyrus, and middle gyrus (98). Total WM injury is increased with the duration of HF (99). These may have negative impacts on cognitive function in HF patients.

A total of two studies (29, 30) have shown the correlation between the reduction of GM volume and the decline of cognitive function in HF patients. However, since the sample size used was small and no control group was present, the universality of the conclusion is limited. Therefore, the changes in GM may be associated with CI in HF patients. However, large-scale prospective studies are required to confirm this hypothesis.

At present, no consistent conclusion has been proposed regarding the relationship between WM changes and cognitive function in HF patients. A total of two studies (26, 28) suggested that WMH was not associated with cognitive function scores in HF patients, while two studies (27, 31) demonstrated that WMH may be mildly or moderately related to cognitive function. The possible causes may be related to a variety of different factors. It was shown that WMH (26) was influenced by several factors, including HF patients with hypertension, DM, AF, and various other diseases, whereas it included higher number of male participants (84%). However, one of these studies included non-demented HF patients, with relatively normal cognitive function, which may also be the reason for the negative results (28). Cerebral WM is affected by several factors, and the complexity of clinical HF patients may also account for the opposite conclusion. Therefore, the changes of WM may not be related to CI in HF patients, which must be confirmed by large-scale prospective studies.

### Effect of LVEF on Cognitive Function in Patients With HF

In terms of the common classification, HF can be classified into the three following groups according to the percentage of EF: HFrEF (EF < 40%), HF with mid-range EF (HFmrEF: 40 ≤ EF < 49%), and HFpEF (EF ≥ 50%). HFmrEF can progress into either HFrEF or HFpEF (100). The decline of LVEF (HFdEF) in HFpEF patients during disease progression is a special type of HF, with grave outcomes (101). HFrEF and HFpEF are the most common types and account for approximately the same proportion in HF populations (102). Certain differences have been noted between HFrEF and HFpEF in the development and progression of the HF disease subtypes. HFrEF is mainly manifested as decreased left ventricular systolic function, and HFpEF is mainly manifested as severe left ventricular diastolic function and increased operative diastolic elastance (ED)/effective arterial elastance (EA), which is more pronounced in women (103). The left ventricular diastolic dysfunction of HFpEF is associated with diffuse myocardial fibrosis (104).

EF is a key factor affecting the progression and prognosis of patients with HF, and it may also affect the cognitive function of patients. Previous studies that investigated EF and CI in HF patients did not reach consistent conclusions, and some of these findings were contradictory. The possible reason for these findings was the average EF level in enrolled HF patients. A total of three studies (38, 43, 47) demonstrated that low EF exhibited adverse effects on certain cognitive domains of HF patients, notably when EF was <30%. The remaining six studies (40–42, 44–46) did not analyze the correlation between EF and cognitive score according to the different range of EF, which may affect the conclusions. The average EF of the study (41) participants was 43.4 ± 15.8%, including HF patients with diastolic dysfunction, which may also affect cognitive function. Therefore, when EF decreases significantly (<30%) in patients with HF, cognitive function may be impaired. When EF > 30%, the effect of EF on cognitive function may not be particularly clear.

Current studies have also yielded inconsistent conclusions regarding the differences in cognitive function among patients with different types of HF. This may be due to the different EF values defined in the EF classification, which were 40% (39), 45% (34, 36), and 50% (35), respectively. The criteria of the three studies (32, 33, 37) were unclear. The inconsistent classification of patients with HF may have a greater impact on the research results. The EF standard of HFrEF was shown to be lower and the damage to cardiovascular and cerebral perfusion was more serious as demonstrated in a previous study (39). In addition, HFpEF patients are often accompanied by diastolic dysfunction, which also damages cognitive function. Therefore, when the defined EF is high, the cognitive scores of HFrEF and HFpEF may be similar.

The possible mechanism is related to the damage of the brain structure and the accumulation of pathological products. The decrease in EF levels may lead to cerebral hypoperfusion, whereas the decrease of EF in patients with HF was significantly correlated with the decrease of GM density in the frontal lobe as well as in the hippocampus and precuneus, which also indicated the existence of cognitive-related brain structure damage (105). In addition, the levels of Neuron Specific Enolase (NSE) are significantly increased in HFrEF patients during the acute decompensated phase, demonstrating an increased rate of SCI (106). It has been proposed that low EF is associated with increased CSF t-tau and p-tau levels in normal cognitive subjects, suggesting that lower EF may lead to neurodegeneration (107). All of these may exacerbate the impairment of cognitive function.

### Effect of Specific Detection Indices on Cognitive Function in Patients With HF

BMI refers to the square of weight/height (kg/m^2^) and is an important international index to measure the degree of obesity. The World Health Organization (WHO) states that normal BMI is between 18.5 and 24.9 kg/m^2^, whereas a BMI value between 25 and 29.9 kg/m^2^ corresponds to overweight subjects, and a BMI ≥ 30 kg/m^2^ is used to characterize obese adults (108).

A total of four studies (24, 37, 49, 50) indicated that BMI was significantly and positively correlated with cognitive function in HF patients. Several possible mechanisms have been proposed for the influence of BMI on the cognition function of HF. Firstly, BMI may affect the brain structure of HF patients. A previous study (109) suggested that a high-fat diet could increase oxidative stress in rats, resulting to damage in the hippocampal neurons. In addition, the increase in BMI interacted with cerebral perfusion, resulting in the decrease of WM and GM (108). A higher BMI value in HF can increase the risk of complications, such as hypertension and T2DM, resulting in poor health, and it may aggravate cognitive dysfunction by affecting endothelial, microvascular, nervous system, and other functions (49). Moreover, BMI and obesity are associated with the changes in the levels of circulating biomarkers, such as brain-derived neurotrophic factor (BDNF), amyloid-beta, and leptin, which can reduce cognitive function in multiple domains (110), notably in attention and executive function (24).

A previous study (51) demonstrated that this association may only apply to male patients. The possible reason was that the obese female patients included in that study were 5.7 years younger than the obese male patients and 8.2 years younger than the non-obese women, and age was a confounding factor affecting the evaluation of the correlation index. Concomitantly, men with more ischemic factors and bad habits would also affect the assessment of the results obtained. A total of two studies (40, 48) reached the opposite conclusion. The lower EF, the more the anxiety and the depression of the participants. Malnutrition or sarcopenia in patients with end-stage HF may be another factor that could explain these findings.

Electrolyte disturbances often occur during the development and treatment of HF, notably with regard to the imbalance of sodium and potassium ions. Excessive intake of sodium leads to the retention of sodium and water, which can aggravate HF. Moreover, since the majority of the patients with HF receive diuretic therapy for a long time period, an imbalance in sodium and potassium can be caused. For example, administration of a loop diuretic for a long period of time can lead to low levels of serum sodium and potassium, while administration of an aldosterone antagonist can increase the content of potassium due to the mechanism of potassium-sparing. The imbalance of sodium and potassium intake is associated with major cardiovascular events and mortality (111).

A total of four studies demonstrated that the high (54) or low (39, 55) sodium and potassium ions as well as the imbalance of sodium/potassium ratio (53) were associated with cognitive decline in HF patients. Several mechanisms may account for these results. High or low blood sodium may affect cognitive function. An *in vivo* study (112) demonstrated that the extracellular glutamate concentration was increased in the hippocampus of rats with chronic hyponatremia, and the sustained low extracellular sodium ions concentration reduced further the glutamate uptake by primary astrocytes, resulting in damage to the long-term enhancement mechanism (LTP). Hyponatremia could also directly affect the distribution of the mitochondria, reducing the content of ATP in neurons. The long-term increase in blood sodium levels will lead to dehydration of brain cells, reduced volume, and decreased antioxidant capacity of the hippocampus (113, 114). Furthermore, blood potassium levels are considered another cause of CI, and insufficient potassium intake may lead to the accumulation of Alzheimer's disease-related pathological products, such as the Aβ protein (115).

However, the levels of urinary sodium were not associated with CI (52). Administration of mixed diuretics could be the main factor affecting patients with severe CI (unable to complete two urine collections, 47% of all participants) although correlation analysis was not performed.

BNP and NT-proBNP are the most promising biomarkers in the clinic, and are mainly used for evaluating cardiac function and the presence of myocardial injury (116). BNP is released from ventricular muscles in response to the increase of ventricular wall stress (117). BNP and NT-proBNP have equally powerful and independent predictive effects on all-cause death and rehospitalization of HF patients (OR 1.46 vs. 1.45) (118). In addition, the increase of BNP and NT-proBNP levels predicts the accelerated decline of cognitive function in the elderly (119). Multiple studies (46, 47, 57, 81–83) have demonstrated that this association may be more significant in patients with HF.

The mechanism of BNP and NT-proBNP affecting cognitive function in patients with HF is unclear. Several possible reasons may account for these findings. BNP reflects left ventricular systolic and diastolic function and is associated with the incidence of additional heart diseases, such as myocardial infarction, AF, valvular disease, and renal function (120). Therefore, BNP may reflect the comprehensive effects of these processes on cognitive function. Higher concentration levels of NT-proBNP were significantly associated with a smaller brain GM volume, which may lead to the decline in cognitive function (121).

### Effects of Comorbidities on Cognitive Function in Patients With HF

HF patients are often accompanied by a variety of other diseases, such as AF, anemia, diabetes, depression, and sleep disorders. These diseases may also affect cognitive function in HF patients.

Depression is very common in HF patients. A previous study indicated that 47.13% of HF patients exhibited depressive symptoms (122). Depression causes more physical symptoms, and increases the hospitalization, mortality, and financial burden of the patients (123, 124).

Previous studies on depression and cognitive dysfunction of HF patients have not reached consistent conclusions, and contradictory results have also been reported. The correlation between cognitive function and depression in HF patients was discovered in 73% of the studies. Furthermore, a previous study (60) indicated that the association between cognitive function and depression in HF patients was mainly driven by non-somatic symptoms of depression. The somatic subscale (three items) exhibited lower number of items compared with that of the non-somatic subscale of PHQ-9 (six items), which may also lead to the correlation between somatic symptoms and cognition.

The underlying mechanism of depression leading to cognitive decline in HF patients is not completely clear. In the present study, several possible reasons may be attributed for these results. It has been shown by various *in vivo* studies that cytokines, such as IL-1 β, IL-6, CX3CR1, and hypocretin-1 are involved in the pathological processes of depression affecting cognition, which may be mediated through direct and indirect effects on long-term enhancement, neurogenesis, and synaptic plasticity (125–127). In contrast to these observations, the occurrence and development of depression may affect cognitive function by leading to deep subcortical WMH, thinning of the hippocampal cortex and abnormal functional connection of the cognitive network (128–130). In addition, depressive symptoms can cause CI, such as attention and executive function in HF patients by exacerbating hypoperfusion, overstimulating the sympathetic nervous system, and reducing mental space, and mental flexibility (131).

However, this association was not found in 27% of studies, and the possible reasons may be multifaceted. In the present study, 55% of HF patients were obese, and the confounding factor was not excluded in the correlation analysis (48). In a previous study (42), the included patients were younger, and 60% of the patients exhibited HF caused by dilated cardiomyopathy, whereas only 1.8% were patients with CI, which may lead to selection bias. The same bias was reported by a previous study (65), which suggested that patients with CI accounted for only 10% of the total sample. However, although MHI was applied to depression assessment (44), the assessment tool was not sensitive enough to capture the extent of the negative mood state, whereas patients with severe CI may lack emotional insight and processing. In summary, these conflicting findings are due to different samples (age of patients, sample size) and to the different instruments used to assess cognitive function, as well as due to the different scales used to evaluate depressive symptoms. Therefore, depression may aggravate CI in patients with HF.

AF is the most common arrhythmia disorder noted in clinical practice, and it is also a cardiovascular disease complication, which is frequently followed by HF. The prevalence of AF in HF patients was 10.6% (132). AF was significantly correlated with cognitive dysfunction in HF patients. A total of five studies (21, 67–70) demonstrated that AF was significantly associated with CI in patients with HF. The mechanism of cognitive decline following AF may be related to cerebral hemodynamic changes caused by irregular rhythm and brain injury caused by microemboli. Arrhythmia and rapid ventricular rate may lead to abnormal changes of CBF volume in AF patients. Saglietto et al. (133) suggested that the higher ventricular rate was associated with a gradual increase in hypoperfusion and hypertensive events by simulating the relationship between ventricular rate and cerebral hemodynamic changes in AF patients. The mechanism of its occurrence may be attributed to irregular ventricular rates in AF patients triggering a higher variability of the distal cerebral hemodynamic variables, which leads to severe cerebral hemodynamic events of reduced blood flow or excessive pressure (134). Moreover, the alteration of microcirculation hemodynamic (pressure and flow rate) patterns during AF may be involved in the occurrence and development of cerebral hypoperfusion (135). Hemodynamic disorders in patients with AF may lead to CI due to chronic ischemia, whereas in the case of brain injury, asymptomatic cerebral infarction may also be a key factor affecting the cognitive function of AF patients. AF was found to be a risk factor for all cortical microinfarcts, and exhibited adverse effects on cognitive function (136). Moreover, Galenko et al. (137) indicated that the expression levels of the biological markers of brain injury, such as astrocyte-specific glial acidic fibrillary protein (GFAP) and microtubule-associated Tau protein were significantly higher in patients with AF than those noted in normal subjects. These studies indicated that brain injury was common in patients with AF. Microthromboembolism derived from the left atrial fibrosis, complex plaque in the aortic arch, or mobile plaques may be important causes of SCI in patients with non-valvular AF (138). These brain injuries contribute to the impairment of cognitive function in AF patients. In addition, the decline of brain autoregulation and connectivity of brain network regions in patients with AF may also lead to the decline of cognitive function. Junejo et al. (139) demonstrated by using specific experiments that patients with AF exhibited impaired neurovascular coupling responses to visual stimulation, while the automatic regulation function of the brain was weakened. Silva et al. (140) demonstrated that the patients with AF exhibited decreased connectivity in regions of the default mode network (DMN) including the frontal lobe (left superior frontal gyrus and left medial frontal gyrus), bilateral precuneus, and left angular gyrus.

However, this study (70) did not yield an association between HF and cognitive function in patients with AF following 1 year of follow-up, which may be due to the short follow-up time period as well as the absence of collection and analysis of the changes in the conditions of HF patients during this period.

DM is the most common endocrine disorder and is also considered to be a common complication of HF. Epidemiological studies have shown that the prevalence of DM ranges between 10 and 47% in the HF cohort, including HFrEF and HFpEF (141). DM is closely related to diastolic dysfunction, decreased quality of life, and increased mortality in HF patients (142, 143). In addition, DM is a significant risk factor for cognitive decline in patients with HF, which has been confirmed by several studies (49, 55, 61, 69, 71, 72). The mechanism of action may be associated with several factors. Hyperglycemia possibly leads to hippocampal tissue damage. An animal study (144) suggested that overexpression of Dipeptidyl peptidase-4 (DPP4) in diabetic rats may reduce peroxisome proliferator-activated receptors γ-Coactivator 1α expression, leading to mitochondrial dysfunction. Hyperglycemia may also increase the levels of inflammatory cytokines, such as interferon-γ (IFN-γ) and interleukin-6 in the hippocampus, resulting in the apoptosis of hippocampal neurons (145). Abnormal expression of vascular endothelial growth factor (VEGF) and insulin resistance in DM patients may also account for the damage of WM as well as the decreased volume of CBF in the hippocampus, occipital lobe, and default model network related areas (146–149). In addition, pathological cerebrovascular neovascularization, increased blood-brain barrier (BBB) permeability, cerebrovascular pericyte dysfunction, and synaptic protein injury may be responsible for the ability of hyperglycemia to affect cognition (150–152).

Anemia is present in more than 50% of HF patients and affects the quality of life and the prognosis of these patients (153, 154). According to WHO, anemia is defined as hemoglobin concentration (Hb) < 130 g/l for adult men, < 120 g/l for adult women, and < 110 g/l for pregnant women. The main causes of anemia in CHF patients are kidney damage, anemia of chronic disease, and iron deficiency. Other factors, including hypothyroidism, deficiency of vitamin B12 and folate are also affecting the development of this disease (155). In the present study, six studies (40, 55, 59, 63, 71, 73) demonstrated that anemia was a risk factor for cognitive deterioration in patients with HF. The possible biological mechanism may be due to brain injury caused by decreased hemoglobin. It has been shown by certain studies that low hemoglobin can cause chronic hypoxia of brain cells, mitochondrial dysfunction, and oxidative stress, resulting in decreased CBF and hippocampal volume, reduced amyloid deposition as well asatrophy of the frontal and temporal cortex (156–161). Anemia is usually caused by iron deficiency or vitamin B12 and folic acid insufficiency. Iron deficiency may induce the decline in cognition, notably in learning and memory function, by decreasing the expression and function of insulin-like growth factors I and II (IGF-I/II) and brain-derived neurotrophic factor (BDNF) in specific areas of the brain (162). The deficiency of vitamin B12 and/or folate can elevate homocysteine levels, which can affect cognitive function by inducing WM damage, brain atrophy, and neurofibrillary tangles (163).

The incidence rate of sleep disorders is high in patients with HF. In a cross-sectional study of 267 HF patients, 61.8% of participants exhibited poor sleep quality (Pittsburgh Sleep Quality Index ≥5) and 22.5% demonstrated excessive daytime sleepiness (Epworth Sleepiness Scale > 10) (75). Poor sleep quality, excessive daytime sleepiness, sleep-disordered breathing (SDB), and insomnia may aggravate cognitive dysfunction in this population.

Among them, daytime sleepiness and SDB have demonstrated significant effects on the cognitive function of HF patients (76–78, 80). It is interesting to note that this study (76) notably emphasized on the significant correlation between cognition and daytime sleepiness in adults with HF (≤ 65). The reason is unclear, and may be related to the fact that adults are more sensitive to body input and sleep drive than the elderly. However, a different study (75) reached the opposite conclusion, possibly due to using self-reported measures of sleep quality and daytime dysfunction, and presenting no objective sleep measure, which may have affected the results.

SDB is a special type of sleep disorder, mainly manifested as abnormal changes in breathing during sleep. It is characterized by repetitive nocturnal apnea and/or hypopnea, followed by a recovery phase with hyperpnea, which leads to circulation patterns of intermittent hypoxia-reoxygenation, arousal, and sympathetic activation (164). A previous study (32) revealed the correlation between cognitive function scores and oxygen desaturation index in HF patients. However, different conclusions have also been reported, possibly due to the use of a portable SDB monitoring device, which may not fully detect the sleep architecture, and the small sample size (*n* = 28) and can also affect the correlation results (74).

The mechanism of sleep disorder affecting cognitive function in HF patients may be based on the dysregulation of the hypothalamic-pituitary-adrenal axis (HPA), which is accompanied by high levels of cortisol and pro-inflammatory factors as well as central cholinergic dysfunction leading to neurotoxicity effects on the hippocampus (165). Alperin et al. (166) has also found that the cortical and subcortical volumes of the bilateral hippocampus and the parietal lobule were significantly reduced in sleep-deprived subjects. Furthermore, sleep disorders may reduce the cerebral flow in the left and right frontal lobes, impair memory consolidation and restore brain function during the night, as well as lead to cognitive decline (167, 168). In addition, daytime sleepiness may be more easily affected by pathological changes related to Alzheimer's disease, such as β-protein deposition in the brain (169). SDB may affect cognitive function due to intermittent cerebral hypoxia, changes in cerebral hemodynamics, and oxygen saturation. These conditions may lead to cognition-related brain GM loss, WM neurons, and neuronal damage (170, 171).

However, the present review contains several limitations. Firstly, the qualitative synthesis rather than the quantitative method was used due to the varied risk factors and the inconsistency of cognitive function evaluation methods. Therefore, specific conclusions could not be drawn with this meta-analysis. Secondly, the majority of the included studies were cross-sectional studies. Therefore, they could not reflect the causal relationship. In the future, large prospective studies are required to verify the relationship between these risk factors and CI in patients with HF.

## Conclusion

A total of 66 literature studies were included to review the imaging changes of the brain and multiple risk factors in patients with CI after HF. The imaging changes included CBF, medial temporal lobe volume, GM volume and WM volume. The risk factors included EF, BNP/NT-proBNP, BMI index, electrolyte disturbance, combined with AF, DM, anemia, depression, sleep disorders and other diseases. Following comprehensive analysis, the following conclusions were obtained: BNP/NT-proBNP and co-morbidities of HF including AF, DM and anemia were inevitably correlated with CI in patients with HF and certain studies obtained independent correlation results. The severity of MTA, GM volume, BMI index, depression, sleep disorder and low EF value (<30%) were inclined to be associated with CI. The reduction in the volume of CBF may be related to CI, whereas the change of the WM volume was likely to be independent of CI in HF patients. The imaging changes and related risk factors summarized in the present study provide a basis for the early warning and prevention of CI following HF, and the clinical attention and improvement of these risk factors in HF patients may alleviate the cognitive decline. In the future, large prospective studies are required to verify the relationship between these risk factors and CI in patients with HF. And we will find the causes of CI in HF patients through study the cerebral perfusion pressure, synaptic function and reactive oxygen species metabolites.

## Data Availability Statement

The original contributions presented in the study are included in the article/Supplementary Material, further inquiries can be directed to the corresponding author/s.

## Author Contributions

YJ and LW: theme and design of the research. ZL, SC, and YT: verification of data. TL and YL: statistical analysis. YJ: writing of the manuscript. MZ and YX: critical revision of the manuscript for intellectual content and obtaining funding. All authors have approved the final manuscript for submission.

## Funding

This study was supported by the National Natural Science Foundation Project of China (no. 81973787) and Beijing Tongzhou District Science and Technology Project (no. KJ2019CX017).

## Conflict of Interest

The authors declare that the research was conducted in the absence of any commercial or financial relationships that could be construed as a potential conflict of interest.

## Publisher's Note

All claims expressed in this article are solely those of the authors and do not necessarily represent those of their affiliated organizations, or those of the publisher, the editors and the reviewers. Any product that may be evaluated in this article, or claim that may be made by its manufacturer, is not guaranteed or endorsed by the publisher.

## Abbreviations

HF: heart failure
CI: cognitive impairment
HFrEF: heart failure with reduced ejection fraction
HFmrEF: heart failure with mid-range ejection fraction
HFpEF: heart failure with preserved ejection fraction
CBF: cerebral blood flow
AF: atrial fibrillation
MTA: medial temporal lobe atrophy
GM: gray matter
WM: white matter
WMH: white matter hyperintensities
EF: ejection fraction
LVEF: left ventricular ejection fraction
BMI: body mass index
BNP: B-type natriuretic peptide
NT-proBNP: N-terminal pro-B-type natriuretic peptide
DM: diabetes mellitus
T2DM: type 2 diabetes mellitus.
