# Supplementary material for: Brain Imaging Changes and Related Risk Factors of Cognitive Impairment in Patients With Heart Failure
Source: Front Cardiovasc Med. 2022 Jan 26;8:838680. doi: 10.3389/fcvm.2021.838680 (PMC8826966; doi:10.3389/fcvm.2021.838680)
Supplement: Supplementary file 1 [file Table_1.DOCX]

Supplementary Material 1

# Retrieval strategy

Search: **((((((((((((((((Heart Failure[Title/Abstract]) OR (Cardiac Failure[Title/Abstract])) OR (Heart Decompensation[Title/Abstract])) OR (Decompensation, Heart[Title/Abstract])) OR (Heart Failure, Right-Sided[Title/Abstract])) OR (Heart Failure, Right Sided[Title/Abstract])) OR (Right-Sided Heart Failure[Title/Abstract])) OR (Right Sided Heart Failure[Title/Abstract])) OR (Myocardial Failure[Title/Abstract])) OR (Congestive Heart Failure[Title/Abstract])) OR (Heart Failure, Congestive[Title/Abstract])) OR (Heart Failure, Left-Sided[Title/Abstract])) OR (Heart Failure, Left Sided[Title/Abstract])) OR (Left-Sided Heart Failure[Title/Abstract])) OR (Left Sided Heart Failure[Title/Abstract])) AND (((((((((((((((((((((((((((Cognitive Dysfunctions[Title/Abstract]) OR (Dysfunction, Cognitive[Title/Abstract])) OR (Dysfunctions, Cognitive[Title/Abstract])) OR (Cognitive Impairments[Title/Abstract])) OR (Cognitive Impairment[Title/Abstract])) OR (Impairment, Cognitive[Title/Abstract])) OR (Impairments, Cognitive[Title/Abstract])) OR (Mild Cognitive Impairment[Title/Abstract])) OR (Cognitive Impairment, Mild[Title/Abstract])) OR (Cognitive Impairments, Mild[Title/Abstract])) OR (Impairment, Mild Cognitive[Title/Abstract])) OR (Impairments, Mild Cognitive[Title/Abstract])) OR (Mild Cognitive Impairments[Title/Abstract])) OR (Mild Neurocognitive Disorder[Title/Abstract])) OR (Disorder, Mild Neurocognitive[Title/Abstract])) OR (Disorders, Mild Neurocognitive[Title/Abstract])) OR (Mild Neurocognitive Disorders[Title/Abstract])) OR (Neurocognitive Disorder, Mild[Title/Abstract])) OR (Neurocognitive Disorders, Mild[Title/Abstract])) OR (Cognitive Decline[Title/Abstract])) OR (Cognitive Declines[Title/Abstract])) OR (Decline, Cognitive[Title/Abstract])) OR (Declines, Cognitive[Title/Abstract])) OR (Mental Deterioration[Title/Abstract])) OR (Deterioration, Mental[Title/Abstract])) OR (Deteriorations, Mental[Title/Abstract])) OR (Mental Deteriorations[Title/Abstract]))) AND ((((((((((((((((((((((((((((((((((((((((((((((((((((((((((((((((((((((((((((((((((((((((((((((((((((((((((((((((((((((((((((((((((((((((((((((((((((((((((((((((((((((((((((((((((((((((((((((((((((((((((((((((((((((((((((((((((((((((((((((((((((((((((((((Brain imaging[Title/Abstract]) OR (Imaging, Brain[Title/Abstract])) OR (Neuroimaging[Title/Abstract])) OR (Risk factors[Title/Abstract])) OR (Factor, Risk[Title/Abstract])) OR (Risk Factor[Title/Abstract])) OR (Health Correlates[Title/Abstract])) OR (Correlates, Health[Title/Abstract])) OR (Risk Scores[Title/Abstract])) OR (Risk Score[Title/Abstract])) OR (Score, Risk[Title/Abstract])) OR (Risk Factor Scores[Title/Abstract])) OR (Risk Factor Score[Title/Abstract])) OR (Score, Risk Factor[Title/Abstract])) OR (Population at Risk[Title/Abstract])) OR (Populations at Risk[Title/Abstract])) OR (Circulation, Cerebrovascular[Title/Abstract])) OR (Cerebral Circulation[Title/Abstract])) OR (Cerebral Circulations[Title/Abstract])) OR (Circulation, Cerebral[Title/Abstract])) OR (Brain Blood Flow[Title/Abstract])) OR (Blood Flow, Brain[Title/Abstract])) OR (Brain Blood Flows[Title/Abstract])) OR (Flow, Brain Blood[Title/Abstract])) OR (Regional Cerebral Blood Flow[Title/Abstract])) OR (Cerebral Perfusion Pressure[Title/Abstract])) OR (Cerebral Perfusion Pressures[Title/Abstract])) OR (Perfusion Pressure, Cerebral[Title/Abstract])) OR (Pressure, Cerebral Perfusion[Title/Abstract])) OR (Cerebral Blood Flow[Title/Abstract])) OR (Blood Flow, Cerebral[Title/Abstract])) OR (Cerebral Blood Flows[Title/Abstract])) OR (Flow, Cerebral Blood[Title/Abstract])) OR (Gray Matters[Title/Abstract])) OR (Matter, Gray[Title/Abstract])) OR (Matters, Gray[Title/Abstract])) OR (Grey Matter[Title/Abstract])) OR (Grey Matters[Title/Abstract])) OR (Matter, Grey[Title/Abstract])) OR (Matters, Grey[Title/Abstract])) OR (Cerebellar Gray Matter[Title/Abstract])) OR (Cerebellar Gray Matters[Title/Abstract])) OR (Gray Matter, Cerebellar[Title/Abstract])) OR (Gray Matters, Cerebellar[Title/Abstract])) OR (Matter, Cerebellar Gray[Title/Abstract])) OR (Matters, Cerebellar Gray[Title/Abstract])) OR (Cerebellar Grey Matter[Title/Abstract])) OR (Cerebellar Grey Matters[Title/Abstract])) OR (Grey Matter, Cerebellar[Title/Abstract])) OR (Grey Matters, Cerebellar[Title/Abstract])) OR (Matter, Cerebellar Grey[Title/Abstract])) OR (Matters, Cerebellar Grey[Title/Abstract])) OR (Hippocampus Proper[Title/Abstract])) OR (Hippocampus Propers[Title/Abstract])) OR (Proper, Hippocampus[Title/Abstract])) OR (Propers, Hippocampus[Title/Abstract])) OR (Ammon Horn[Title/Abstract])) OR (Horn, Ammon[Title/Abstract])) OR (Ammon's Horn[Title/Abstract])) OR (Ammons Horn[Title/Abstract])) OR (Horn, Ammon's[Title/Abstract])) OR (Cornu Ammonis[Title/Abstract])) OR (Hippocampal Formation[Title/Abstract])) OR (Formation, Hippocampal[Title/Abstract])) OR (Formations, Hippocampal[Title/Abstract])) OR (Hippocampal Formations[Title/Abstract])) OR (Schaffer Collaterals[Title/Abstract])) OR (Schaffer Collateral[Title/Abstract])) OR (Collateral, Schaffer[Title/Abstract])) OR (Collaterals, Schaffer[Title/Abstract])) OR (Subiculum[Title/Abstract])) OR (Subiculums[Title/Abstract])) OR (Lobe, Temporal[Title/Abstract])) OR (Lobes, Temporal[Title/Abstract])) OR (Temporal Lobes[Title/Abstract])) OR (Temporal Region[Title/Abstract])) OR (Region, Temporal[Title/Abstract])) OR (Regions, Temporal[Title/Abstract])) OR (Temporal Regions[Title/Abstract])) OR (Temporal Cortex[Title/Abstract])) OR (Cortex, Temporal[Title/Abstract])) OR (Cortices, Temporal[Title/Abstract])) OR (Temporal Cortices[Title/Abstract])) OR (Inferior Horn of the Lateral Ventricle[Title/Abstract])) OR (Temporal Horn[Title/Abstract])) OR (Horn, Temporal[Title/Abstract])) OR (Horns, Temporal[Title/Abstract])) OR (Temporal Horns[Title/Abstract])) OR (Inferior Horn of Lateral Ventricle[Title/Abstract])) OR (Temporal Horn of the Lateral Ventricle[Title/Abstract])) OR (Planum Polare[Title/Abstract])) OR (Planum Polares[Title/Abstract])) OR (Polare, Planum[Title/Abstract])) OR (Polares, Planum[Title/Abstract])) OR (Temporal Sulcus[Title/Abstract])) OR (Sulcus, Temporal[Title/Abstract])) OR (Superior Temporal Gyrus[Title/Abstract])) OR (Gyrus, Superior Temporal[Title/Abstract])) OR (Temporal Gyrus, Superior[Title/Abstract])) OR (Gyrus Temporalis Superior[Title/Abstract])) OR (Gyrus Temporalis Superiors[Title/Abstract])) OR (Superior, Gyrus Temporalis[Title/Abstract])) OR (Superiors, Gyrus Temporalis[Title/Abstract])) OR (Temporalis Superior, Gyrus[Title/Abstract])) OR (Temporalis Superiors, Gyrus[Title/Abstract])) OR (Temporal Gyrus[Title/Abstract])) OR (Gyrus, Temporal[Title/Abstract])) OR (Temporal Operculum[Title/Abstract])) OR (Operculum, Temporal[Title/Abstract])) OR (Operculums, Temporal[Title/Abstract])) OR (Temporal Operculums[Title/Abstract])) OR (Fusiform Gyrus[Title/Abstract])) OR (Gyrus, Fusiform[Title/Abstract])) OR (Lateral Occipito-Temporal Gyrus[Title/Abstract])) OR (Gyrus, Lateral Occipito-Temporal[Title/Abstract])) OR (Lateral Occipito Temporal Gyrus[Title/Abstract])) OR (Occipito-Temporal Gyrus, Lateral[Title/Abstract])) OR (Lateral Occipitotemporal Gyrus[Title/Abstract])) OR (Gyrus, Lateral Occipitotemporal[Title/Abstract])) OR (Occipitotemporal Gyrus, Lateral[Title/Abstract])) OR (Occipitotemporal Gyrus[Title/Abstract])) OR (Gyrus, Occipitotemporal[Title/Abstract])) OR (Gyrus Fusiformis[Title/Abstract])) OR (Fusiformi, Gyrus[Title/Abstract])) OR (Fusiformis, Gyrus[Title/Abstract])) OR (Gyrus Fusiformi[Title/Abstract])) OR (Matter, White[Title/Abstract])) OR (Matters, White[Title/Abstract])) OR (White Matters[Title/Abstract])) OR (Cerebellar White Matter[Title/Abstract])) OR (Cerebellar White Matters[Title/Abstract])) OR (Matter, Cerebellar White[Title/Abstract])) OR (Matters, Cerebellar White[Title/Abstract])) OR (White Matter, Cerebellar[Title/Abstract])) OR (White Matters, Cerebellar[Title/Abstract])) OR (Ejection fraction[Title/Abstract])) OR (EF[Title/Abstract])) OR (Electrolyte[Title/Abstract])) OR (potassium[Title/Abstract])) OR (sodium[Title/Abstract])) OR (Body mass index[Title/Abstract])) OR (BMI[Title/Abstract])) OR (Obesity[Title/Abstract])) OR (Atrial Fibrillations[Title/Abstract])) OR (Fibrillation, Atrial[Title/Abstract])) OR (Fibrillations, Atrial[Title/Abstract])) OR (Auricular Fibrillation[Title/Abstract])) OR (Auricular Fibrillations[Title/Abstract])) OR (Fibrillation, Auricular[Title/Abstract])) OR (Fibrillations, Auricular[Title/Abstract])) OR (Persistent Atrial Fibrillation[Title/Abstract])) OR (Atrial Fibrillation, Persistent[Title/Abstract])) OR (Atrial Fibrillations, Persistent[Title/Abstract])) OR (Fibrillation, Persistent Atrial[Title/Abstract])) OR (Fibrillations, Persistent Atrial[Title/Abstract])) OR (Persistent Atrial Fibrillations[Title/Abstract])) OR (Familial Atrial Fibrillation[Title/Abstract])) OR (Atrial Fibrillation, Familial[Title/Abstract])) OR (Atrial Fibrillations, Familial[Title/Abstract])) OR (Familial Atrial Fibrillations[Title/Abstract])) OR (Fibrillation, Familial Atrial[Title/Abstract])) OR (Fibrillations, Familial Atrial[Title/Abstract])) OR (Paroxysmal Atrial Fibrillation[Title/Abstract])) OR (Atrial Fibrillation, Paroxysmal[Title/Abstract])) OR (Atrial Fibrillations, Paroxysmal[Title/Abstract])) OR (Fibrillation, Paroxysmal Atrial[Title/Abstract])) OR (Fibrillations, Paroxysmal Atrial[Title/Abstract])) OR (Paroxysmal Atrial Fibrillations[Title/Abstract])) OR (Anemia[Title/Abstract])) OR (Anemias[Title/Abstract])) OR (Depression[Title/Abstract])) OR (Depressions[Title/Abstract])) OR (Depressive Symptoms[Title/Abstract])) OR (Depressive Symptom[Title/Abstract])) OR (Symptom, Depressive[Title/Abstract])) OR (Symptoms, Depressive[Title/Abstract])) OR (Emotional Depression[Title/Abstract])) OR (Depression, Emotional[Title/Abstract])) OR (Depressions, Emotional[Title/Abstract])) OR (Emotional Depressions[Title/Abstract])) OR (Sleep Disorders[Title/Abstract])) OR (Disorder, Sleep Wake[Title/Abstract])) OR (Disorders, Sleep Wake[Title/Abstract])) OR (Sleep Wake Disorder[Title/Abstract])) OR (Wake Disorder, Sleep[Title/Abstract])) OR (Wake Disorders, Sleep[Title/Abstract])) OR (Subwakefullness Syndrome[Title/Abstract])) OR (Subwakefullness Syndromes[Title/Abstract])) OR (Syndrome, Subwakefullness[Title/Abstract])) OR (Syndromes, Subwakefullness[Title/Abstract])) OR (Sleep Disorders[Title/Abstract])) OR (Disorder, Sleep[Title/Abstract])) OR (Disorders, Sleep[Title/Abstract])) OR (Sleep Disorder[Title/Abstract])) OR (Sleep-Related Neurogenic Tachypnea[Title/Abstract])) OR (Neurogenic Tachypnea, Sleep-Related[Title/Abstract])) OR (Neurogenic Tachypneas, Sleep-Related[Title/Abstract])) OR (Sleep Related Neurogenic Tachypnea[Title/Abstract])) OR (Sleep-Related Neurogenic Tachypneas[Title/Abstract])) OR (Tachypnea, Sleep-Related Neurogenic[Title/Abstract])) OR (Tachypneas, Sleep-Related Neurogenic[Title/Abstract])) OR (Long Sleeper Syndrome[Title/Abstract])) OR (Long Sleeper Syndromes[Title/Abstract])) OR (Sleeper Syndrome, Long[Title/Abstract])) OR (Sleeper Syndromes, Long[Title/Abstract])) OR (Syndrome, Long Sleeper[Title/Abstract])) OR (Syndromes, Long Sleeper[Title/Abstract])) OR (Short Sleeper Syndrome[Title/Abstract])) OR (Short Sleeper Syndromes[Title/Abstract])) OR (Sleeper Syndrome, Short[Title/Abstract])) OR (Sleeper Syndromes, Short[Title/Abstract])) OR (Syndrome, Short Sleeper[Title/Abstract])) OR (Syndromes, Short Sleeper[Title/Abstract])) OR (Short Sleep Phenotype[Title/Abstract])) OR (Phenotype, Short Sleep[Title/Abstract])) OR (Phenotypes, Short Sleep[Title/Abstract])) OR (Short Sleep Phenotypes[Title/Abstract])) OR (Sleep Phenotypes, Short[Title/Abstract])) OR (BNP[Title/Abstract])) OR (Peptide, Brain Natriuretic[Title/Abstract])) OR (BNP-32[Title/Abstract])) OR (BNP 32[Title/Abstract])) OR (Brain Natriuretic Peptide-32[Title/Abstract])) OR (Brain Natriuretic Peptide 32[Title/Abstract])) OR (Natriuretic Peptide-32, Brain[Title/Abstract])) OR (Peptide-32, Brain Natriuretic[Title/Abstract])) OR (Natriuretic Factor-32[Title/Abstract])) OR (Natriuretic Factor 32[Title/Abstract])) OR (BNP Gene Product[Title/Abstract])) OR (Type-B Natriuretic Peptide[Title/Abstract])) OR (Natriuretic Peptide, Type-B[Title/Abstract])) OR (Type B Natriuretic Peptide[Title/Abstract])) OR (Natriuretic Peptide Type-B[Title/Abstract])) OR (Natriuretic Peptide Type B[Title/Abstract])) OR (Nesiritide[Title/Abstract])) OR (Brain Natriuretic Peptide[Title/Abstract])) OR (B-Type Natriuretic Peptide[Title/Abstract])) OR (Natriuretic Peptide, B-Type[Title/Abstract])) OR (Ventricular Natriuretic Peptide, B-type[Title/Abstract])) OR (Ventricular Natriuretic Peptide, B type[Title/Abstract])) OR (Natrecor[Title/Abstract])) OR (N-terminal pro-BNP[Title/Abstract])) OR (proBNP(1-76)[Title/Abstract])) OR (NTproBNP[Title/Abstract])) OR (proBNP (1-76)[Title/Abstract])) OR (N-BNP peptide[Title/Abstract])) OR (NT-BNP[Title/Abstract])) OR (Amino-terminal pro-brain natriuretic peptide[Title/Abstract])) OR (aminoterminal pro-B-type natriuretic peptide[Title/Abstract])) OR (NT-proBNP[Title/Abstract])) OR (Brain Derived Neurotrophic Factor[Title/Abstract])) OR (Factor, Brain-Derived Neurotrophic[Title/Abstract])) OR (Neurotrophic Factor, Brain-Derived[Title/Abstract])) OR (BDNF[Title/Abstract]))** Sort by: **Most Recent**
