# Supplementary material for: Brain Imaging Changes and Related Risk Factors of Cognitive Impairment in Patients With Heart Failure
Source: Front Cardiovasc Med. 2022 Jan 26;8:838680. doi: 10.3389/fcvm.2021.838680 (PMC8826966; doi:10.3389/fcvm.2021.838680)
Supplement: Supplementary file 2 [file Table_2.DOCX]

Supplementary Material 2

Table 1. The bias of included cross-sectional studies according to AHRQ tool

| Study ID | QI | Q2 | Q3 | Q4 | Q5 | Q6 | Q7 | Q8 | Q9 | Q10 | Q11 | Total score |
| --- | --- | --- | --- | --- | --- | --- | --- | --- | --- | --- | --- | --- |
| Leeuwis AE 2020 (18) | 1 | 1 | 1 | 1 | 1 | 1 | 1 | 1 | 0 | 0 | 0 | 8 |
| Suzuki H 2016 (19) | 1 | 1 | 1 | 0 | 1 | 1 | 1 | 1 | 0 | 0 | 0 | 7 |
| Kure CE 2016 (20) | 1 | 1 | 0 | 0 | 1 | 1 | 1 | 1 | 0 | 0 | 0 | 6 |
| Alosco ML 2015 (21) | 1 | 1 | 0 | 0 | 1 | 1 | 0 | 1 | 0 | 1 | 0 | 6 |
| Alosco ML 2013 (23) | 1 | 1 | 0 | 1 | 1 | 1 | 0 | 1 | 0 | 0 | 0 | 6 |
| Alosco ML 2012 (24) | 1 | 1 | 0 | 1 | 1 | 1 | 0 | 1 | 0 | 0 | 0 | 6 |
| Jesus PA 2006 (25) | 1 | 1 | 0 | 1 | 1 | 1 | 0 | 0 | 0 | 0 | 0 | 5 |
| Frey A 2018 (26) | 1 | 1 | 0 | 0 | 1 | 1 | 1 | 1 | 0 | 1 | 0 | 7 |
| Vogels RL 2007 (28) | 1 | 1 | 0 | 0 | 1 | 1 | 1 | 1 | 0 | 1 | 0 | 7 |
| Alosco ML 2016 (29) | 1 | 1 | 0 | 0 | 1 | 1 | 1 | 0 | 0 | 1 | 0 | 6 |
| Alosco ML 2013 (30) | 1 | 1 | 0 | 0 | 1 | 1 | 1 | 1 | 0 | 1 | 1 | 8 |
| Alosco ML 2013 (31) | 1 | 1 | 0 | 1 | 1 | 1 | 0 | 1 | 0 | 0 | 0 | 6 |
| Faulkner KM 2020 (33) | 1 | 1 | 1 | 0 | 1 | 1 | 1 | 0 | 0 | 0 | 0 | 6 |
| Warraich Haider J 2018 (36) | 1 | 1 | 1 | 1 | 1 | 1 | 0 | 1 | 0 | 0 | 0 | 7 |
| Adebayo PB 2017 (37) | 1 | 1 | 1 | 0 | 1 | 1 | 1 | 1 | 0 | 1 | 0 | 8 |
| Shin MS 2017 (38) | 1 | 1 | 0 | 0 | 1 | 1 | 0 | 1 | 0 | 1 | 0 | 6 |
| Albabtain M 2016 (39) | 1 | 1 | 1 | 1 | 1 | 1 | 0 | 1 | 0 | 0 | 0 | 7 |
| Graham S 2014 (40) | 1 | 1 | 1 | 0 | 1 | 1 | 1 | 1 | 0 | 1 | 0 | 8 |
| Feola M 2013 (41) | 1 | 1 | 1 | 1 | 1 | 1 | 1 | 1 | 0 | 1 | 0 | 9 |
| Steinberg Gerrit 2011 (42) | 1 | 1 | 0 | 0 | 1 | 1 | 1 | 1 | 0 | 1 | 0 | 7 |
| Festa Joanne R 2011 (43) | 1 | 1 | 1 | 0 | 1 | 1 | 1 | 1 | 0 | 0 | 0 | 7 |
| Hoth Karin F 2008 (45) | 1 | 1 | 0 | 0 | 1 | 1 | 0 | 1 | 0 | 1 | 0 | 6 |
| Feola M 2007 (46) | 1 | 1 | 1 | 0 | 1 | 1 | 0 | 1 | 0 | 1 | 0 | 7 |
| Zuccalà G 1997 (47) | 1 | 1 | 1 | 0 | 1 | 1 | 1 | 0 | 0 | 1 | 0 | 7 |
| Ely AV 2020 (48) | 1 | 1 | 0 | 0 | 1 | 1 | 0 | 1 | 0 | 0 | 0 | 5 |
| Alosco ML 2015 (49) | 1 | 1 | 0 | 1 | 1 | 1 | 0 | 1 | 0 | 1 | 0 | 7 |
| Hawkins MA 2013 (51) | 1 | 1 | 0 | 0 | 1 | 1 | 1 | 1 | 0 | 0 | 0 | 6 |
| Dolansky MA 2016 (52) | 1 | 1 | 0 | 0 | 1 | 1 | 0 | 1 | 0 | 0 | 0 | 5 |
| Hwang SY 2016 (53) | 1 | 1 | 0 | 0 | 1 | 1 | 1 | 1 | 0 | 1 | 0 | 7 |
| Alosco ML 2013 (54) | 1 | 1 | 0 | 1 | 1 | 1 | 0 | 1 | 0 | 1 | 0 | 7 |
| Zuccalà G 2005 (55) | 1 | 1 | 1 | 0 | 1 | 1 | 0 | 1 | 0 | 0 | 0 | 6 |
| Sargent L 2020 (56) | 1 | 1 | 0 | 0 | 1 | 1 | 0 | 1 | 0 | 0 | 0 | 5 |
| Pierobon A2020 (57) | 1 | 1 | 0 | 0 | 1 | 1 | 1 | 1 | 0 | 0 | 0 | 6 |
| Lee JK 2018 (58) | 1 | 1 | 1 | 0 | 1 | 1 | 1 | 1 | 0 | 0 | 0 | 7 |
| Taraghi Z 2016 (59) | 1 | 1 | 1 | 0 | 1 | 1 | 0 | 1 | 0 | 0 | 0 | 6 |
| Hawkins MA 2015 (60) | 1 | 1 | 0 | 0 | 1 | 1 | 0 | 1 | 0 | 0 | 0 | 5 |
| Hanon O 2014 (62) | 1 | 1 | 1 | 0 | 1 | 1 | 1 | 1 | 0 | 0 | 0 | 7 |
| Pulignano G 2014 (63) | 1 | 1 | 0 | 0 | 1 | 1 | 1 | 1 | 0 | 0 | 1 | 7 |
| Garcia S 2011 (64) | 1 | 1 | 0 | 0 | 1 | 1 | 0 | 1 | 0 | 0 | 0 | 5 |
| Akomolafe A 2005 (65) | 1 | 1 | 0 | 1 | 1 | 1 | 0 | 1 | 0 | 0 | 0 | 6 |
| Wei-Jia WANG 2020 (67) | 1 | 1 | 0 | 0 | 1 | 1 | 0 | 1 | 1 | 0 | 1 | 7 |
| Yang H 2017 (68) | 1 | 1 | 1 | 1 | 1 | 1 | 1 | 1 | 0 | 0 | 0 | 8 |
| Coma M 2016 (69) | 1 | 1 | 1 | 0 | 1 | 1 | 0 | 1 | 0 | 0 | 0 | 6 |
| Pulignano G 2016 (70) | 1 | 1 | 0 | 1 | 1 | 1 | 0 | 1 | 0 | 0 | 1 | 7 |
| Basile G 2013 (71) | 1 | 1 | 1 | 1 | 1 | 1 | 0 | 1 | 0 | 0 | 0 | 7 |
| Alosco ML 2012 (72) | 1 | 1 | 0 | 1 | 1 | 1 | 1 | 1 | 0 | 0 | 0 | 7 |
| Kim EY 2019 (73) | 1 | 1 | 0 | 0 | 1 | 1 | 0 | 1 | 0 | 0 | 0 | 5 |
| Moon C 2018 (74) | 1 | 1 | 0 | 0 | 1 | 1 | 0 | 1 | 0 | 0 | 0 | 5 |
| Walter FA 2018 (75) | 1 | 1 | 0 | 0 | 1 | 1 | 0 | 1 | 0 | 0 | 0 | 5 |
| Byun E 2017 (76) | 1 | 1 | 0 | 0 | 1 | 1 | 0 | 1 | 0 | 0 | 0 | 5 |
| Moon C 2017 (77) | 1 | 1 | 1 | 0 | 1 | 1 | 0 | 1 | 0 | 0 | 0 | 6 |
| Moon C 2015 (78) | 1 | 1 | 0 | 0 | 1 | 1 | 1 | 1 | 0 | 1 | 0 | 7 |
| Hjelm C 2013 (79) | 1 | 1 | 0 | 0 | 1 | 1 | 0 | 1 | 0 | 0 | 0 | 5 |
| Dong Y 2019 (81) | 1 | 1 | 0 | 1 | 1 | 1 | 0 | 1 | 0 | 0 | 0 | 6 |
| Leto L 2015 (82) | 1 | 1 | 1 | 1 | 1 | 1 | 0 | 1 | 0 | 0 | 1 | 8 |

Q1, Define the source of imformation (survey, record review); Q2, List inclusion and exclusion criteria for exposed and unexposed subjects (cases and controls) or refer to previous publications; Q3, Indicate time period used for identifying patients; Q4, Indicate whether or not subjects were consecutive if not population-based; Q5, Indicate if evaluators of subjective components of study were masked to other aspects of the status of the participants; Q6, Describe any assessments undertaken for quality assurance purposes (e.g., testing/retesting of primary outcome measurements); Q7, Explain any patients exclusions from analysis; Q8, Describe how confounding was assessed and/or controlled; Q9, If applicable, explain how missing data were handled in the analysis; Q10, Summarize patients response rates and completeness of data collection; Q11, Clarify what follow-up, if any, was expected and the percentage of patients for which incomplete data or follow-up was obtained.

Table 2: The bias of included case-control studies according to NOS tool

| Study ID | QI | Q2 | Q3 | Q4 | Q5 | Q6 | Q7 | Q8 | Total score |
| --- | --- | --- | --- | --- | --- | --- | --- | --- | --- |
| Beer C 2009 (27) | 1 | 1 | 1 | 1 | 1 | 1 | 1 | 0 | 7 |
| Almeida O P 2013 (31) | 1 | 0 | 1 | 1 | 1 | 1 | 1 | 1 | 7 |
| D'Elia Emilia 2021 (32) | 1 | 0 | 0 | 1 | 1 | 1 | 1 | 0 | 5 |
| Sauvé MJ 2009(44) | 1 | 0 | 1 | 1 | 1 | 1 | 1 | 0 | 6 |
| Hjelm C 2014 (61) | 1 | 1 | 0 | 1 | 1 | 1 | 1 | 0 | 6 |
| Trojano L 2003 (66) | 1 | 0 | 0 | 1 | 1 | 1 | 1 | 0 | 5 |

Q1, Is the case definition adequate?; Q2, Representativeness of the cases; Q3, Selection of Controls; Q4, Definition of Controls; Q5, Comparability of cases and controls on the basis of the design or analysis; Q6, Ascertainment of exposure; Q7, Same method of ascertainment for cases and controls; Q8, Non-Response rate.

Table 3 The bias of included cohort studies according to NOS tool

| Study ID | QI | Q2 | Q3 | Q4 | Q5 | Q6 | Q7 | Q8 | Total score |
| --- | --- | --- | --- | --- | --- | --- | --- | --- | --- |
| Hammond CA 2018 (34) | 1 | 1 | 1 | 1 | 1 | 1 | 1 | 1 | 8 |
| Witt Lucy S 2018 (35) | 1 | 1 | 1 | 1 | 1 | 1 | 1 | 1 | 8 |
| Riegel B 2011 (80) | 1 | 1 | 1 | 1 | 1 | 1 | 0 | 0 | 6 |

Q1, Representativeness of the exposed cohort; Q2, Selection of the non exposed cohort; Q3, Ascertainment of exposure; Q4, Demonstration that outcome of interest was not present at start of study; Q5, Comparability of cohorts on the basis of the design or analysis; Q6, Assessment of outcome;; Q7, Was follow-up long enough for outcomes to occur; Q8,: Adequacy of follow up of cohorts.

Table 4: The bias of included self control studies according to MINORS tool

| Study ID | QI | Q2 | Q3 | Q4 | Q5 | Q6 | Q7 | Q8 | Q9 | Q10 | Q11 | Q12 | Total score |
| --- | --- | --- | --- | --- | --- | --- | --- | --- | --- | --- | --- | --- | --- |
| Alosco ML 2014 (22) | 2 | 1 | 0 | 2 | 0 | 2 | 2 | 0 | 0 | 0 | 0 | 0 | 9 |
| Alosco ML 2014 (50) | 2 | 0 | 0 | 2 | 0 | 2 | 2 | 0 | 0 | 0 | 0 | 0 | 8 |
| van Vliet P 2014 (83) | 2 | 1 | 0 | 2 | 0 | 2 | 2 | 0 | 0 | 0 | 0 | 0 | 9 |

Q1, a clearly stated aim; Q2, inclusion of consecutive patients; Q3, prospective collection of data; Q4, endpoints appropriate to the aim of the study; Q5, unbiased assessment of the study endpoint; Q6, follow-up period appropriate to the aim of the study; Q7, loss to follow-up less than 5%; Q8, prospective calculation of the study size; Q9, an adequate control group; Q10, contemporary groups; Q11, baseline equivalence of groups; Q12, adequate statistical analyse
